# Landscape in the gallbladder mycobiome and bacteriome of patients undergoing cholelithiasis with chronic cholecystitis

**DOI:** 10.3389/fmicb.2023.1131694

**Published:** 2023-03-22

**Authors:** Junqing Hu, Jichao Tang, Xinpeng Zhang, Kaijin Yang, Ayan Zhong, Qin Yang, Yanjun Liu, Yi Li, Tongtong Zhang

**Affiliations:** ^1^Center of Gastrointestinal and Minimally Invasive Surgery, Department of General Surgery, The Third People’s Hospital of Chengdu, Affiliated Hospital of Southwest Jiaotong University, The Second Affiliated Hospital of Chengdu, Chongqing Medical University, Chengdu, China; ^2^The Center for Obesity and Metabolic Health, The Third People’s Hospital of Chengdu, Affiliated Hospital of Southwest Jiaotong University, The Second Affiliated Hospital of Chengdu, Chongqing Medical University, Chengdu, China; ^3^Medical Research Center, The Third People’s Hospital of Chengdu, Affiliated Hospital of Southwest Jiaotong University, The Second Affiliated Hospital of Chengdu, Chongqing Medical University, Chengdu, China; ^4^General Surgery Day Ward, Department of General Surgery, The Third People’s Hospital of Chengdu, Affiliated Hospital of Southwest Jiaotong University, The Second Affiliated Hospital of Chengdu, Chongqing Medical University, Chengdu, China; ^5^Section for Hepato-Pancreato-Biliary Surgery, Department of General Surgery, The Third People’s Hospital of Chengdu, Affiliated Hospital of Southwest Jiaotong University, The Second Affiliated Hospital of Chengdu, Chongqing Medical University, Chengdu, China

**Keywords:** gallstone disease, marker gene sequencing, microbiota, bile and gallstone, bacteria, fungi

## Abstract

Gallstone disease (GSD) is associated with changes in the gut and gallbladder bacterial composition, but there is limited information on the role of the fungal community (mycobiome) in disease development. This study aimed to characterize the gallbladder mycobiome profiles and their interactions with bacteriome in GSD. A total of 136 bile and gallstone samples (34 paired for bacteriome, and 33 paired and extra 2 bile samples for mycobiome) were obtained from calculi patients with chronic cholecystitis. Bile and gallstone bacteriome and mycobiome were profiled by 16S and internal transcribed spacer (ITS) rRNA gene sequencing, respectively. Gallbladder bacteriome, mycobiome, and interkingdom and intrakingdom interactions were compared between bile and gallstone. In general, microbial diversity was higher in bile than in gallstone, and distinct microbial community structures were observed among them. Deep Sea Euryarchaeotic Group, Rhodobacteraceae, and Rhodobacterales were microbial biomarkers of bile, while Clostridiales and *Eubacterium coprostanoligenes* were biomarkers of gallstone. Five fungal taxa, including *Colletotrichum*, *Colletotrichum sublineola*, and *Epicoccum*, were enriched in gallstone. Further ecologic analyses revealed that intensive transkingdom correlations between fungi and bacteria and intrakingdom correlations within them observed in gallstone were significantly decreased in bile. Large and complex fungal communities inhabit the gallbladder of patients with GSD. Gallstone, compared with bile, is characterized by significantly altered bacterial taxonomic composition and strengthened bacterial–bacterial, fungal–fungal, and bacterial–fungal correlations in the gallbladder of patients with GSD.

## Introduction

Gallstone disease (GSD) has been prevalent worldwide, especially in Western countries and China in the last decades (Fan et al., 2017; Scherber et al., 2017). Most of the gallstones in the gallbladder are the cholesterol type (approximately two-thirds), and the remaining are mainly pigment stones (Portincasa and Wang, 2012). It was previously suggested that a healthy human biliary system is sterile; however, several years ago, it was recognized that the gallbladder has a complex microbiota in non-pathological conditions (Verdier et al., 2015; Warburton et al., 2017). Despite the high worldwide prevalence of GSD, the role of the biliary microbiota in gallstone pathogenesis remains unclear. To date, knowledge about the composition of the biliary microbiota and its influence on the development of biliary disease is limited. Previous studies have linked biliary infection with gallstone development and indicated that bacteria may act as the nucleating factor initiating the formation of both pigment and cholesterol gallstones (Maki, 1966; Swidsinski and Lee, 2001; Stewart et al., 2002; Begley et al., 2005; Stewart et al., 2006). The presence of living bacteria in gallstones has been demonstrated using multiple methods (Swidsinski et al., 1995; Lee et al., 1999; Hazrah et al., 2004; Ramana Ramya et al., 2017). Moreover, several studies have shown that the alterations of bacteria in bile were linked to biliary diseases, such as cholelithiasis (Petrov et al., 2020), cholangiocarcinoma (Chen B. et al., 2019), common bile duct stones (Choe et al., 2021; Kim et al., 2021), biliary injury (Ying Liu et al., 2018), and primary sclerosing cholangitis (Pereira et al., 2017; Timur Liwinski et al., 2019). While works have reported that bacteria could be detected in gallstones, the bacterial spectrum remains unclear in cholelithiasis.

Fungi, as eukaryotes, are ancestrally and ecologically intrinsic to terrestrial life with multiple roles, extending to the regulobiotic. Notably, fungal species have been reported to colonize as commensals in many niches in healthy humans (Cui et al., 2013). Alterations within the fungi are associated with different diseases. The association between fungi and gastrointestinal disease has been well documented, with a special focus on candidiasis (Mukherjee et al., 2015). Studies performed in the past decade have demonstrated that fungi have a complex, multifaceted role in the gastrointestinal tract and actively and directly influence health and disease. A recent retrospective study found that *Candida* presented in bile at a low level (1.3%) by culture (Serra et al., 2021). However, the fungal organisms in bile and gallstone remain largely unclear. Fungi may have the potential to manipulate neighboring bacterial communities or vice versa. Therefore, interactions between the mycobiome and the bacteriome may also play a role in GSD.

In light of the above, we investigated both the fungal and bacterial profiles in paired bile and gallstone samples from patients with cholelithiasis using ITS and 16S rRNA gene high-throughput sequencing. To the best of our knowledge, this is the first study to uncover the fungal spectrum and its interaction with bacteria in both bile and gallstone. Thus, the current study provides insights into mycobiome in GSD. Importantly, the bile origin of the gallstone microbiota and fungal–bacterial interactions might contribute to the stone formation of cholelithiasis.

## Materials and methods

### Sample collection and processing

The stone and bile samples from gallbladder-stone patients were obtained during laparoscopic cholecystectomy. In total, 136 samples, 68 (34 paired bile and gallstone samples) for bacterial analysis and 68 (33 paired bile and gallstone samples, and 2 bile samples only) for fungal analysis, were acquired from 35 patients (Table 1, Supplementary Table S1). The gallstones were classified as cholesterol gallstones based on their physical characteristics (smooth, round to ovoid and yellow-white and laminated or crystalline cut surface).

**Table 1:** Demographic and Clinical Details of GSD Subjects.

Data	Factor	Bile	Gallstone	#Paired
16S of bacteria	Sample size	34	34	34
	Age,^a^ year	43 ± 14	43 ± 14	
	Male^b^	11 (32.4%)	11 (32.4%)	
	Cholesterol^b^	29 (85.3%)	29 (85.3%)	
ITS of fungi	Sample size	35	33	33
	Age,^a^ year	43 ± 14	42 ± 13	
	Male^b^	11 (31.4%)	11 (33.3%)	
	Cholesterol^b^	30 (85.7%)	28 (84.8%)	

^a^Mean ± standard deviation (SD).

^b^Count (percentage).

### Extraction of microbial DNA from bile and gallstone

The total microbial genomic DNA extraction from bile samples (200 μL) was performed by QIAamp DNA Mini Kit (QIAGEN, Germany) following the manufacturer’s instructions. Similarly, microbial DNA from gallstone (approximately 200 mg) was collected using QIAamp Fast DNA Stool Mini Kit (QIAGEN, Germany) according to the handbook. DNA concentrations were detected using a NanoDrop 2000 (Thermo Fisher Scientific, Waltham, MA, USA). DNA samples were stored at −80°C until required for experiments.

### Determination of bacterial and fungal profiles by amplicon sequencing

The V3–V4 region of the bacterial 16S rRNA gene was amplified from extracted DNA using the 341F (CCTAYGGGRBGCASCAG) and 806R (GGACTACNNGGGTATCTAAT) primer sets. For fungal analysis, ITS1 variable region was amplified with universal primers ITS1F (CTTGGTCATTTAGAGGAAGTAA) and ITS2 (GCTGCGTTCTTCATCGATGC). 16S amplicon sequencing was performed on the Illumina MiSeq platform (Applied Protein Technology Co., Ltd., Shanghai, China). ITS amplicon sequencing was conducted by OE Biotech Co., Ltd. (Shanghai, China).

### Data processing

The sequencing data of bacteria and fungi were imported into QIIME 2 (v2022.2) for preprocessing, denoising, diversity analyses and taxonomy classification (Bolyen et al., 2019). The amplicon sequence variants (ASVs) < 0.005% were finally removed from the analysis of both bacterial and fungal data. For the taxonomic assignment, the SILVA 138 and UNITE v8.3 datasets were used for bacteria and fungi, respectively. The QIIME artifacts were inputted into R by the file2meco package^1^, followed by the analysis and plotting using the microeco package (v0.9.0) in R 4.1.0 (Liu et al., 2021). FUNGuild database was used for fungal data to identify fungal guilds by microeco. The Tax4Fun2 (v1.1.5) workflow was applied for the prediction of metagenome functions of bacteria (Wemheuer et al., 2020).

### Co-occurrence network analysis

The general networks of bacteria–bacteria and fungi–fungi in gallbladder were constructed using SparCC correlation through microeco in R. The global network of bacteria–fungi in the gallbladder was generated using SparCC correlation by integrated Network Analysis Pipeline [iNAP, (Feng et al., 2022)]. Networks were also constructed for bacteria–bacteria, fungi–fungi, and bacteria–fungi in bile and gallstone separately by using this platform. Correlated genus pairs were selected when the absolute values of sparse correlation were |*r*| > 0.1 and *p* < 0.05. Visualization and analysis of the network were conducted using Gephi (v0.9.2) (Bastian et al., 2009).

### Statistical analysis

The nonparametric Kruskal Wallis and Permutational multivariate analysis of variance (PERMANOVA, 999 permutations) were used to test the difference among groups of microbial alpha and beta diversity separately. Differentially abundant taxa were identified by linear discriminant analysis (LDA) effect size (LEfSe). In network analysis, pseudo *p*-values were calculated *via* a bootstrap procedure. First, shuffled (w. replacement) datasets were created, and then the SparCC correlation for each dataset was computed. Finally, the two-sided *p*-values were computed based on the correlation results. A chi-square (χ^2^) test was used to compare the difference in the number of correlations between bile and gallstone. Two-sided *t* test was applied to compare the difference in the relative abundance of taxa between bile and gallstone. The differences in predicted function outcomes of bacteria among the groups were compared using the STAMP software v2.1.3.^2^

## Results

### Clinical characteristics of study subjects

The study included 35 subjects: 29 cholesterol subjects and 6 pigment subjects (Table 1, Supplementary Table S1). For bacteriological analysis, the median age was 43 years for subjects from both the bile and gallstone groups. For mycological analysis, the median age was 43 years for the bile group and 42 years for the gallstone group. Other clinical characteristics (sex and type of stone) were comparable between subjects of bile and gallstone groups. In total, 4,518 features were obtained using the bacterial 16S rRNA metagenomic sequencing and 2,431 features were obtained using fungal ITS1 metagenomic sequencing.

### Largely different bacterial community in bile and gallstone of GSD

**Figure 1:**
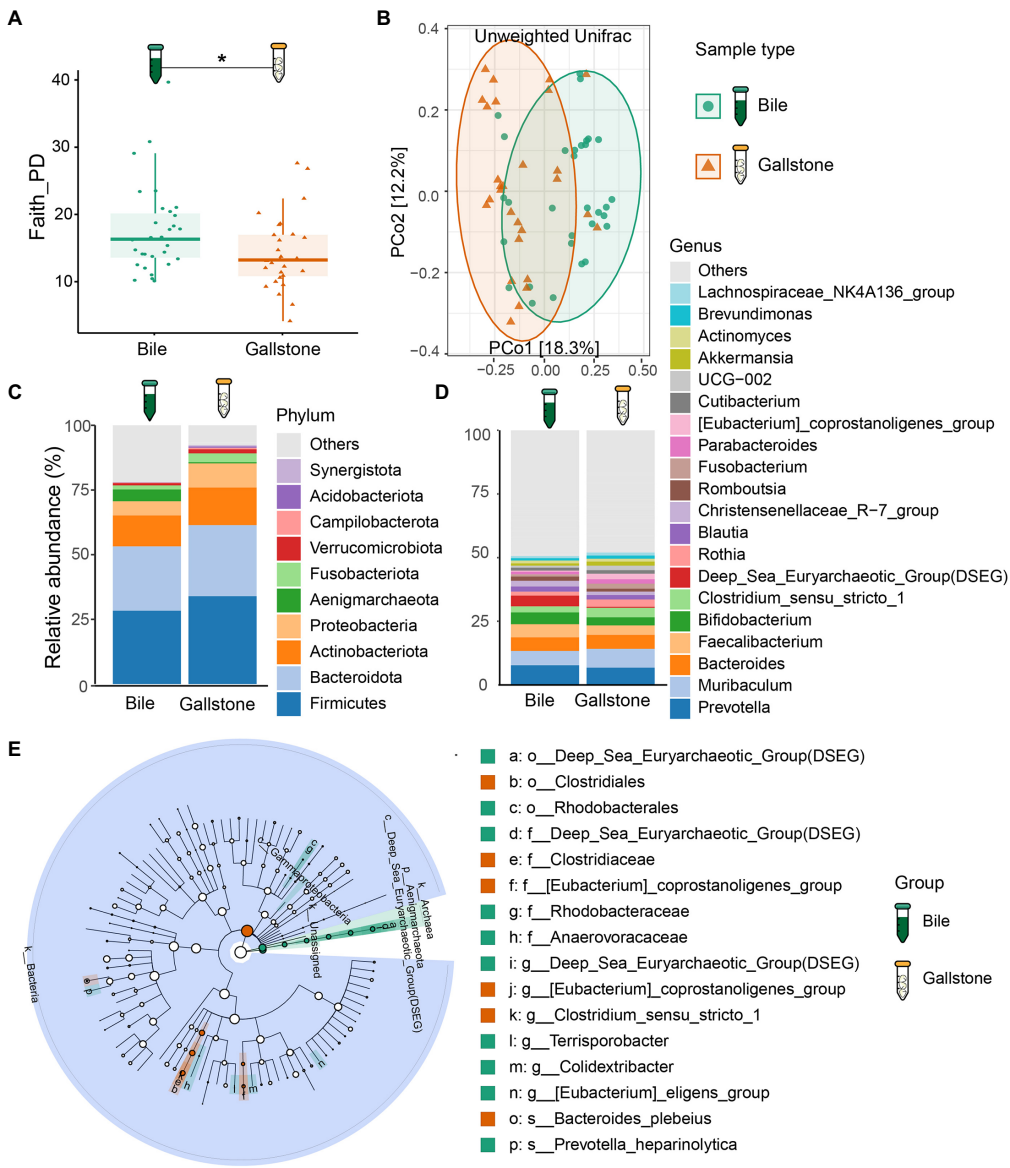
Bacteriome signature of bile and gallstone from GSD patients with chronic cholecystitis. **(A)** Boxplot showed the bacterial phylogenetic diversity (Faith PD) between bile (*n* = 31) and gallstone (*n* = 28). Statistic was performed by a Kruskal-Wallis (pairwise) test. *P (= 0.028) < 0.05. **(B)** Principal coordinate analysis (PCoA) of the unweighted Unifrac distance between bile (*n* = 31) and gallstone (*n* = 28). Statistic was performed by a pairwise PERMANOVA test (999 permutations; *p* = 0.001, *q* = 0.001). **(C,D)** Bar plot depicted the bacterial mean relative abundance at the phylum **(C)** and genus **(D)** levels in both bile and gallstone samples. **(E)** LEfSe cladogram showed the different abundance of bacteria between bile and gallstone. The diameter of each circle was proportional to its abundance (LDA score > 3.0).

First, we compared the bacterial diversity and composition between bile and gallstone. Although bile and gallstone showed similar Shannon diversity and Observed features (ASVs) (Supplementary Figure S1), the Faith phylogenetic diversity (Faith PD, *p* = 0.028) of bile was markedly higher than that of gallstone (Figure 1A). Principal coordinates analysis (PCoA) of unweighted Unifrac distance showed distinct clustering of bile and gallstone samples (Figure 1B; Supplementary Figure S2). Furthermore, it is known that age and sex have an effect on the human microbiota. Thus, we explored the age, sex as well as the gallstone type whether affect the microbial diversity in this study. There was no significant correlation between age and alpha diversity indices (Supplementary Figures S3A,B). In addition, no significant difference in alpha diversity between men and women (Supplementary Figures S3C,D), cholesterol and pigment stones (Supplementary Figures S3E,F) in this study was observed. These results suggest that age, sex, and stone type have negligible effects on the gallbladder bacteriome profile. These results suggest that age, sex, and stone type have negligible effects on the gallbladder bacteriome profile.

Based on 16S rRNA gene sequencing, the relative abundance of bacteria fluctuated largely in bile samples (27.55–98.50%), whereas it accounted for 66.53 to 100% in gallstone samples. Among the bacterial phyla, Firmicutes, Bacteroidetes, Actinobacteria, and Proteobacteria were the four dominant bacterial phyla in the gallbladder bacteriome (Figure 1C; Supplementary Figure S4A). At the genus level, *Muribaculum*, *Bacteroides*, *Prevotella*, *Faecalibacterium*, and *Bifidobacterium* were the dominant bacterial genera in gallbladder bacteriome (Figure 1D; Supplementary Figure S4B). Furthermore, the archaea also accounted for a large proportion. An unknown archaea phylum and Aenigmarchaeota were the major dominant archaea.

**Figure 2:**
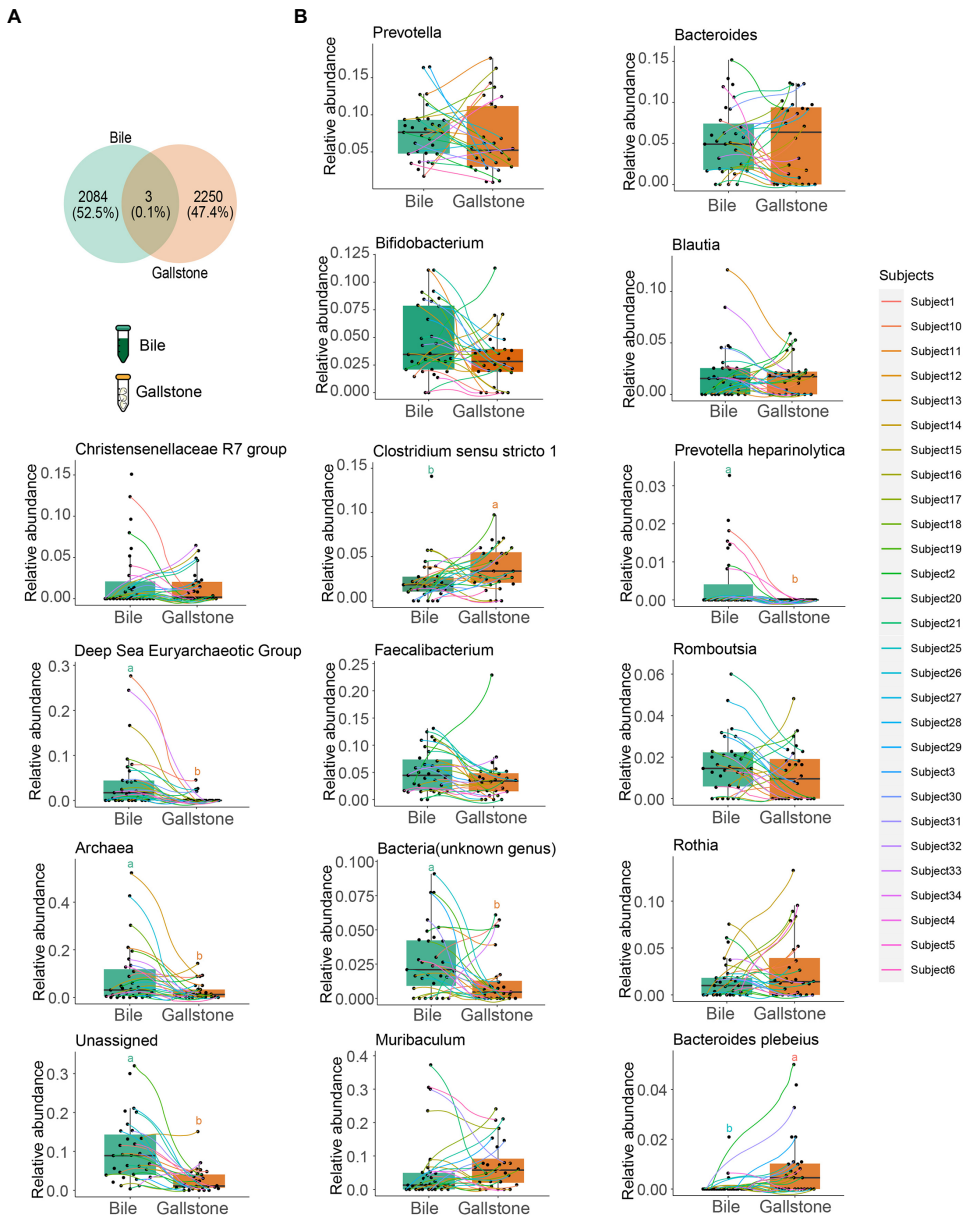
Comparation of the bacterial taxonomic composition between bile and gallstone from GSD patients with chronic cholecystitis. **(A)** Venn diagram of unique and shared ASVs among bile and gallstone samples. The percentage data is the sequence number/total sequence number. **(B)** Relative abundance of microbiota between bile (*n* = 31) and gallstone (*n* = 28) samples at multiple levels. Statistic was performed by a two-sided *t* test. Different lowercase letters indicated significant differences (*p* < 0.05).

We further accessed the bacterial signatures associated with gallstone by LEfSe and random forest (RF). After setting the LDA cutoff score at 3.0, we identified 10 and 6 taxa to be enriched in bile and gallstone, respectively. The Aenigmarchaeota presented a pronounced growth in bile samples (Figure 1E; Supplementary Figure S5). Deep Sea Euryarchaeotic Group (DSEG), Rhodobacteraceae, and Rhodobacterales were microbial biomarker in bile samples, whereas *Eubacterium coprostanoligenes* was a biomarker in gallstone samples, as indicated by both LEfSe and RF outcomes (Figure 1E; Supplementary Figure S5). Additionally, *Clostridiales*, Clostridiaceae, and *Bacteroides plebeius* was enriched in gallstone, whereas *Prevotella heparinolytica* was enriched in bile (Figures 1E, 2B; Supplementary Figure S5A).

As presented in Figure 2A, the detected bacterial sequences between bile and gallstone were almost completely different, only sharing 0.1%. Then, we explored the difference in bacterial composition in paired bile and gallstone samples (Figure 2B). The highest relative abundance of genus *Muribaculum* was increased from bile to gallstone. *Prevotella* was lower, and *Bacteroides* was higher in the gallstone than in bile. *Faecalibacterium* and *Bifidobacterium* were decreased in gallstone. The *Clostridium sensu stricto* 1, an unknown genus, Archaea, and unassigned bacteria also had a significant change from bile to gallstone. Taken together, these results suggest different bacterial phylogenetic diversity and taxonomic composition between bile and gallstone.

### Similar fungal community in bile and gallstone of GSD

**Figure 3:**
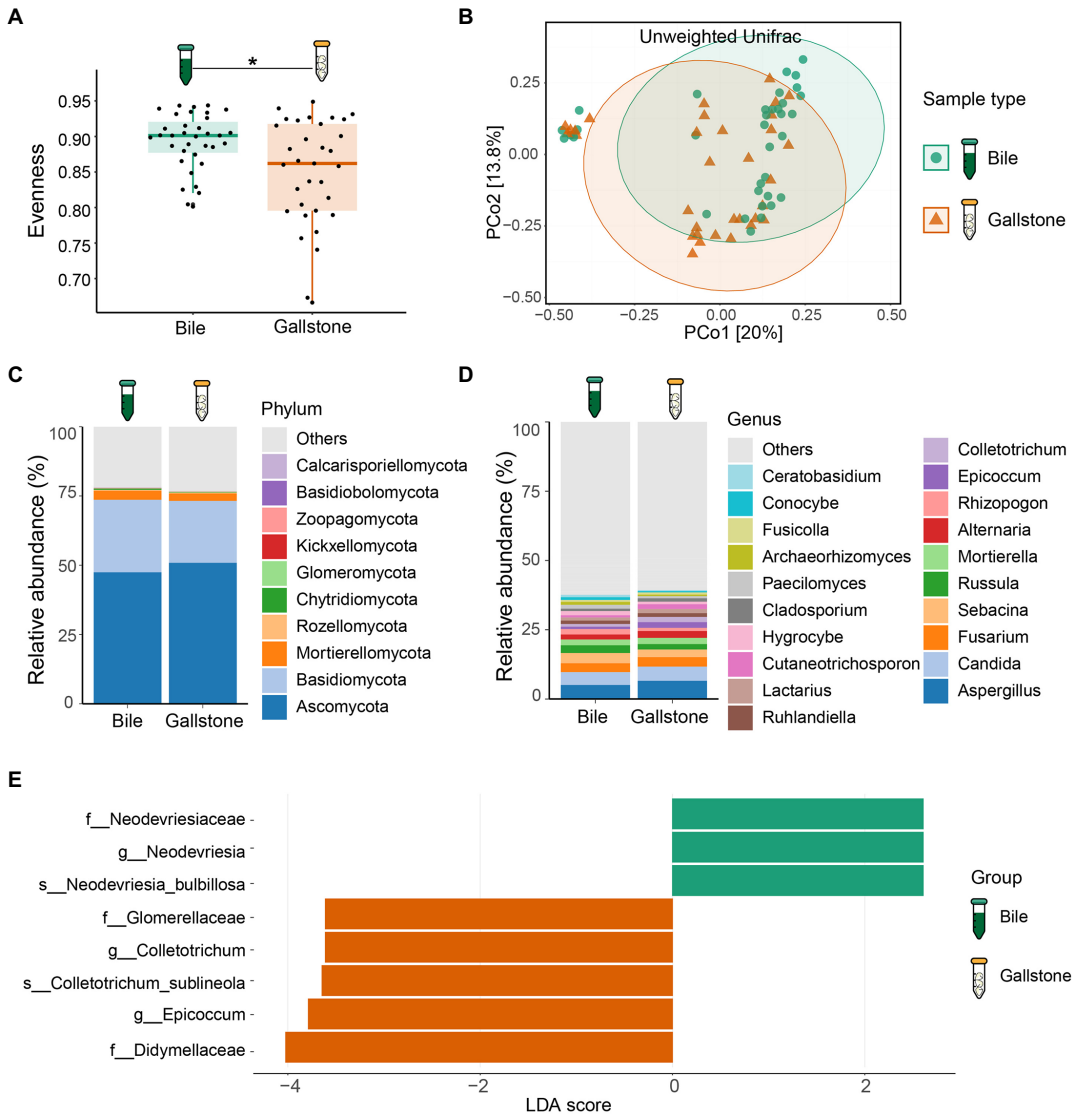
Mycobiome profile of bile and gallstone from GSD patients with chronic cholecystitis. **(A)** Boxplot presented the fungal community evenness between bile (*n* = 35) and gallstone (*n* = 33). Statistic was performed by a Kruskal-Wallis (pairwise) test. *P (= 0.018) < 0.05. **(B)** Principal coordinate analysis (PCoA) of the unweighted Unifrac distance between bile (*n* = 35) and gallstone (*n* = 33). Statistic was performed by a pairwise PERMANOVA test (999 permutations; *p* = 0.038, *q* = 0.038). **(C,D)** Bar plot depicted the fungal mean relative abundance at the phylum **(C)** and genus **(D)** levels in both bile and gallstone samples. **(E)** LEfSe barplot showed the different abundance of fungi between bile and gallstone (LDA score > 2.0).

To study the difference in gallbladder mycobiome in bile and gallstone, we first explored fungal alpha diversity indices between them. We found decreased evenness (Pielou’s e) of the gallbladder mycobiome in gallstone samples compared with bile samples (*p* = 0.0018, Figure 3A). However, there were no changes in fungal diversity (Shannon) and richness (Observed ASVs) between the gallstone and bile samples (Supplementary Figure S6). PCoA based on the unweighted Unifrac distance between individual mycobiome revealed that the gallbladder mycobiome composition of bile and gallstone samples was separated into two distinct clusters, suggesting a different trend of gallbladder mycobiome profiles between bile and stone (*p* = 0.038; Figure 3B; Supplementary Figure S7). Similarly, there was no significant correlation/difference between age, sex, and stone type and alpha diversity indices (Supplementary Figure S8), suggesting that age, sex, and stone type have negligible effects on the gallbladder bacteriome profile.

**Figure 4:**
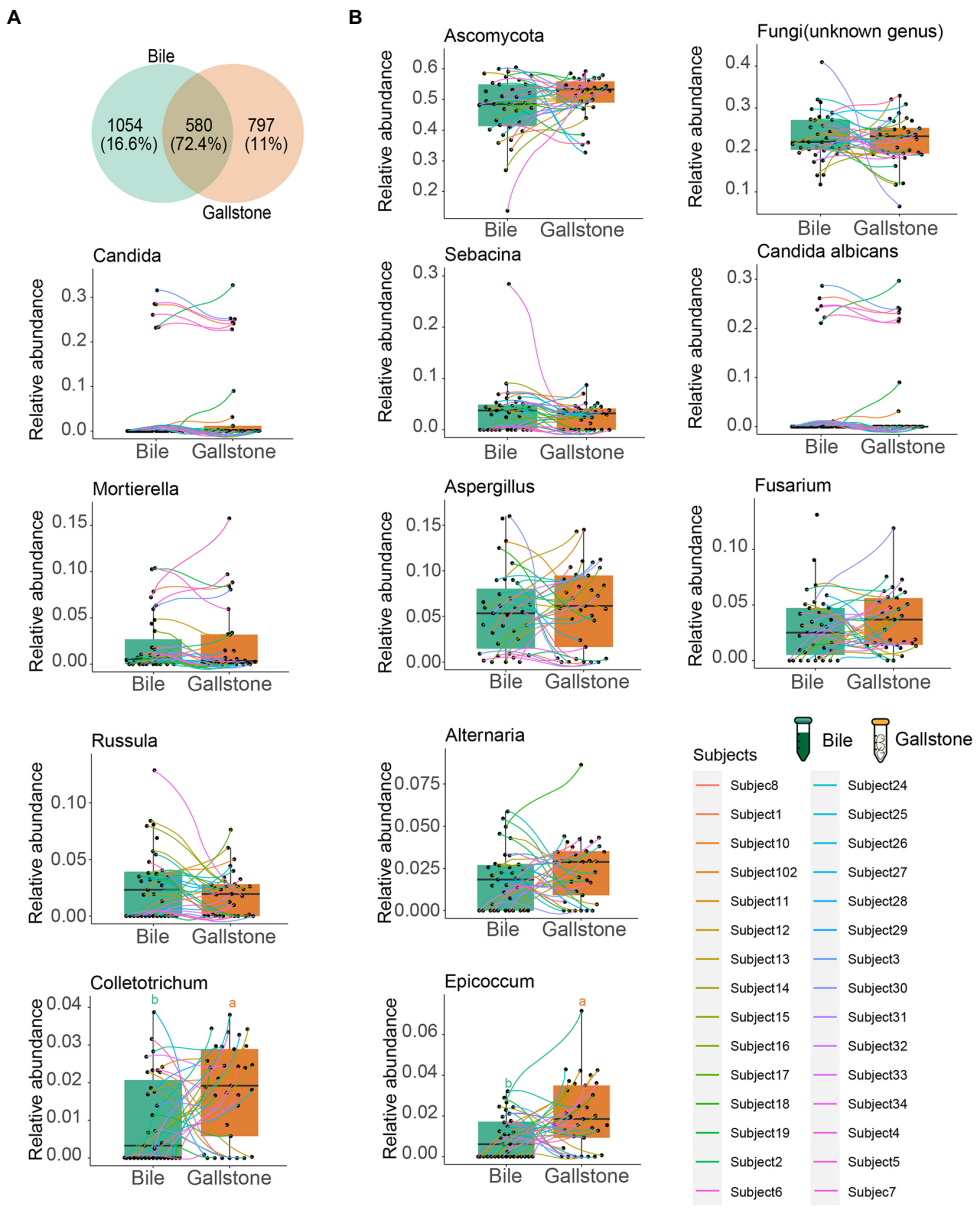
Comparation of the fungal taxonomic composition between bile and gallstone from GSD patients with chronic cholecystitis. **(A)** Venn diagram of unique and shared ASVs among bile and gallstone samples. The percentage data is the sequence number/total sequence number. **(B)** Relative abundance of microbiota between bile (*n* = 35) and gallstone (*n* = 33) samples at multiple levels. Statistic was performed by a two-sided *t* test. Different lowercase letters indicated significant differences (*p* < 0.05).

At the phylum level, Ascomycota, Basidiomycota and Mortierellomycota dominated the gallbladder mycobiome (Figure 3C; Supplementary Figure S9A). Among the genera, *Aspergillus*, *Candida*, *Fusarium*, *Sebacina*, *Russula*, *Mortierella*, *Alternaria*, *Rhizopogon*, *Epicoccum*, and *Colletotrichum* were the dominant fungal genera in the gallbladder mycobiome (Figure 3D; Supplementary Figure S9B). Furthermore, LEfSe (LDA = 2.0) identified five fungal signatures associated with gallstone (Figure 3E). Among the differential taxa, *Colletotrichum* and *Epicoccum* had very small relative abundance (Figure 4B). The species *Colletotrichum sublineola* was also enriched in gallstone.

We also further explored the similarity of fungal composition between bile and gallstone. More than 70% (72.4%) shared fungal ASVs between bile and gallstone were observed (Figure 4A). In addition, phylum Ascomycota, the largest relative abundance genera *Candida*, *Sebacina*, an unknown genus, *Mortierella*, *Aspergillus*, *Fusarium*, and *Russula*, and species *Candida albicans* had no difference between bile and gallstone (Figure 4B). These results imply a similar fungal diversity and taxonomic composition between bile and gallstone.

### Co-occurrence of bacteria, fungi and bacteria–fungi in the gallbladder of GSD

To explore the microbial keystone taxa in the gallbladder, we constructed microbial co-occurrence networks using significant pairwise correlations between microbial taxa, bacteria-bacteria, fungi**–**fungi and bacteria**–**fungi. In general, we found 6 modules (M1**–**6) and four key communities (keystone taxa: *Muribaculum*, *Clostridium sensu stricto* 1, *Prevotella*, and *DSEG*) in bacteria of the gallbladder (Supplementary Figure S10A); six modules (M1**–**6) included four independent communities in fungi of the gallbladder (Supplementary Figure S10B). For the bacteria**–**fungi network (Supplementary Figure S10C; Supplementary Table S2), we obtained a network of four modules consisting of both bacteria (17 taxa) and fungi (23 taxa).

**Figure 5:**
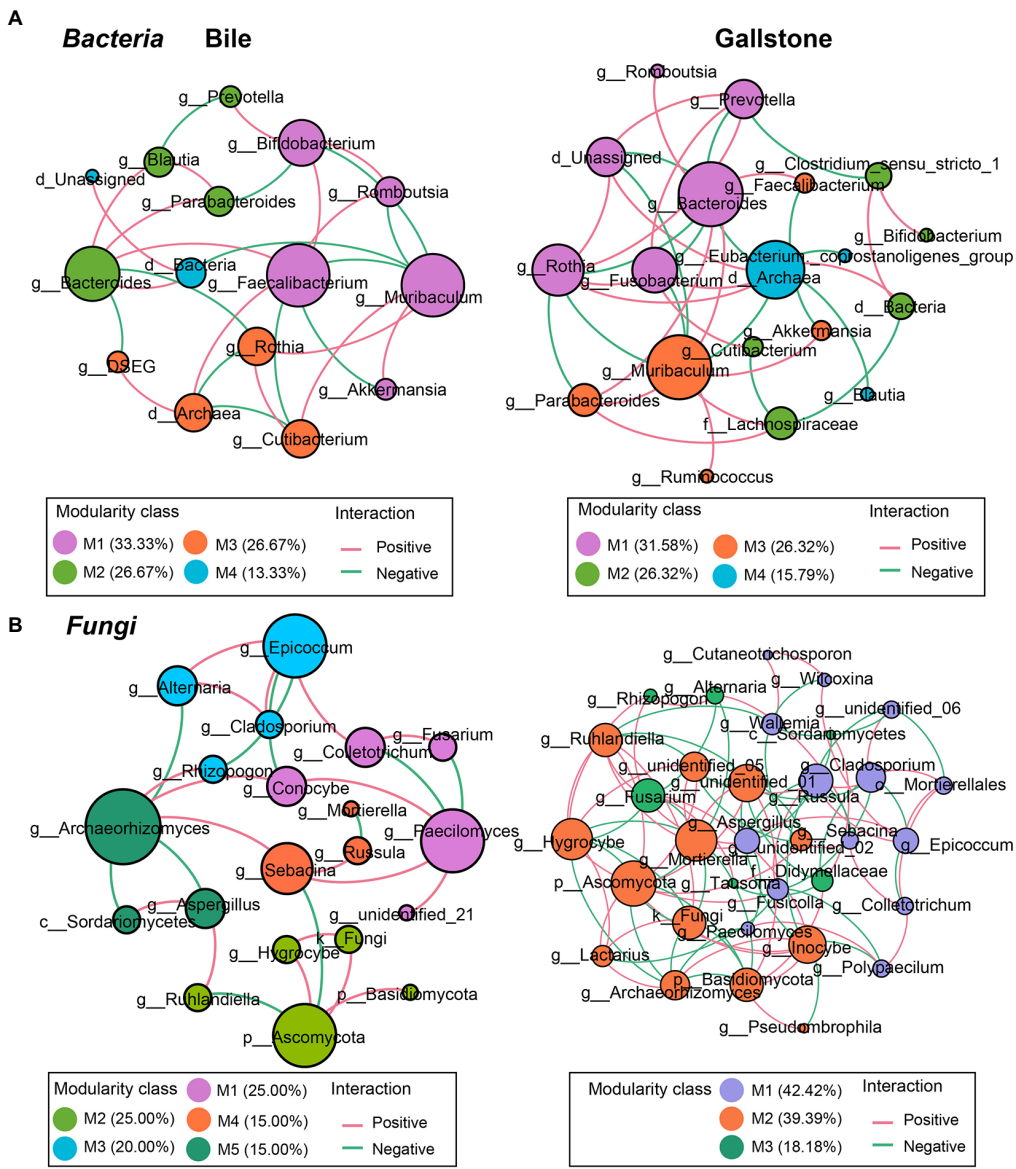
Co-occurrence networks of the intrakingdom correlations of bacteriome and mycobiome in gallbladder of GSD patients with chronic cholecystitis. **(A,B)** Bacterial–bacterial **(A)** and fungal–fungal **(B)** co-occurrence networks of bile (left) and gallstone (right) at the genus level. Node size was presented by its degree (3–15). “M” represented the module of the network.

To understand the network complexity and keystone taxa in gallstone, we compared the bacteria**–**bacteria, fungi**–**fungi, and bacteria**–**fungi co-occurrence networks between bile and gallstone. Although there were four modules of bacterial network found in both bile and gallstone, the network in gallstone had more correlations than that in bile (37 vs. 28; χ^2^ test, *p* = 0.047; Figure 5A; Table 2; Supplementary Tables S3, S4). The keystone taxa were *Muribaculum*, *Faecalibacterium*, *Bifidobacterium*, *Bacteroides*, *Rothia*, and *Cutibacterium* in bile whereas *Bacteroides*, *Muribaculum*, Archaea, *Rothia*, *Fusobacterium*, *Prevotella*, and *Lachnospiraceae* in gallstone (Figure 5A). For fungi**–**fungi interactions, five modules (keystone taxa: *Paecilomyces*, Ascomycota, *Epicoccum*, *Sebacina*, and *Archaeorhizomyces*) were observed in bile, whereas only three modules (keystone taxa: Ascomycota, *Fusarium*, and *Russula*) were found in gallstone. However, the correlations of the network in gallstone were more than three times those in bile, primarily driven by an increased number of nodes (94 vs. 28; χ^2^ test, *p* = 1.50E-37; Figure 5B; Table 2; Supplementary Tables S5, S6).

**Table 2:** Summary of bacterial-bacterial, fungal-fungal and bacterial-fungal co-occurrence networks in bile and gallstone.

Network	Sample	#Node	#Edge	Positive (%)	Negative (%)	*χ*^2^ for #edge	Positive/Negative
Bacteria–Bacteria	Bile	15	28	57.14	42.86	0.047	1.33
	Gallstone	19	37	56.76	43.24		1.31
Fungi–Fungi	Bile	20	28	64.29	35.71	1.50E-37	1.80
	Gallstone	33	94	51.06	48.94		1.04
Bacteria–Fungi	Bile	34	94	64.89	35.11	3.88E-72	1.85
	Gallstone	46	267	55.81	44.19		1.26

**Figure 6:**
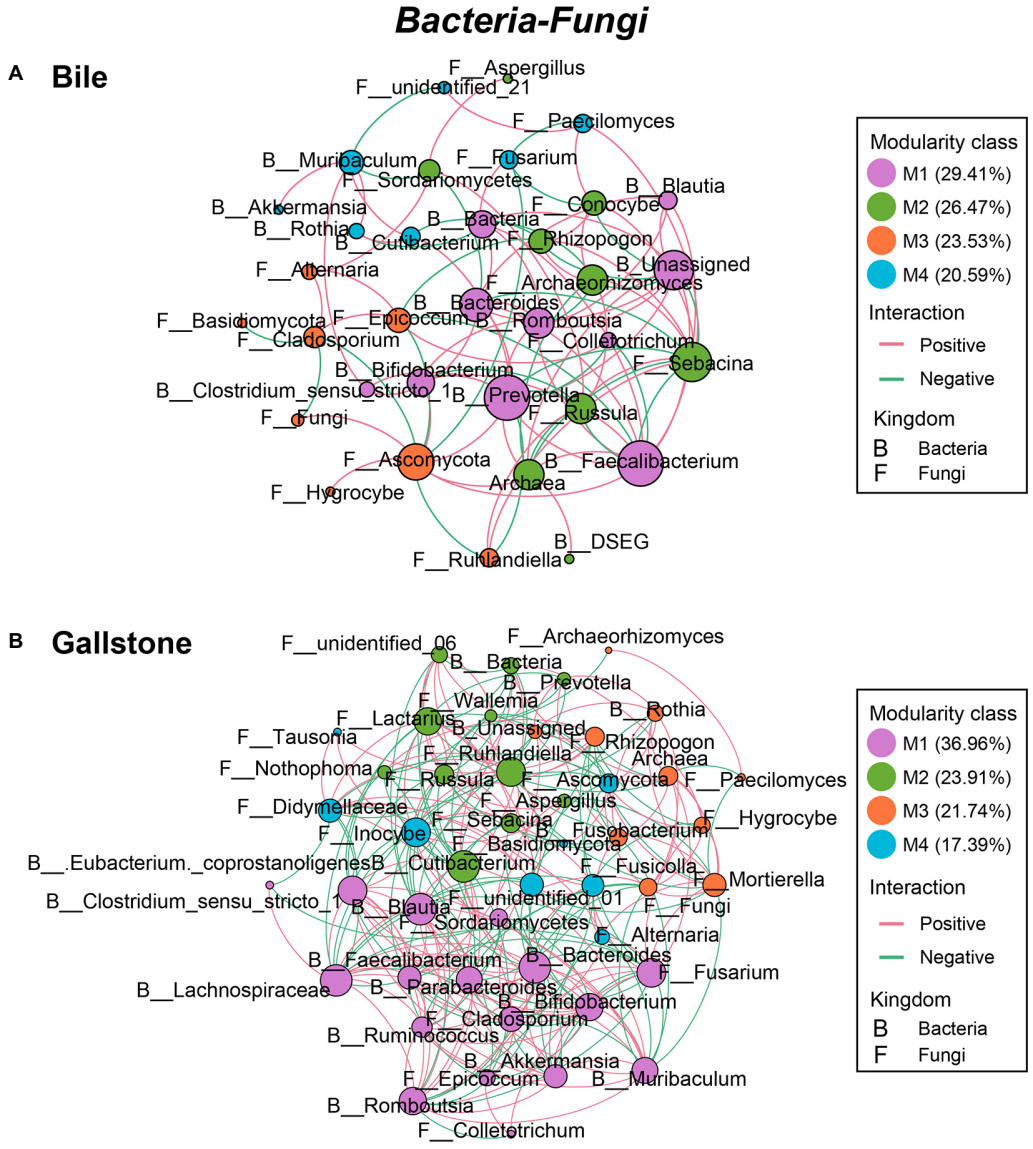
Co-occurrence networks of the interkingdom correlations between bacteriome and mycobiome in gallbladder of GSD patients with chronic cholecystitis. **(A,B)** Bacterial–fungal co-occurrence networks of bile **(A)** and gallstone **(B)** at the genus level. Node size was presented by its degree (3–15). “M” represented the module of the network.

Similarly, the network of bacteria**–**fungi interactions in gallstone was also greater than in bile, despite that they both had four modules (267 vs. 94; χ^2^ test, *p* = 3.88E-72; Figures 6A,B; Table 2; Supplementary Tables S7, S8). Bacteria *Faecalibacterium*, *Prevotella*, *Bacteroides*, *Romboutsia*, and Unassigned, and fungi *Sebacina*, Ascomycota, *Archaeorhizomyces*, *Epicoccum*, and others were key taxa in bile (Figure 6A). Bacteria Lachnospiraceae, *Muribaculum*, *Bacteroides*, *Blautia*, *Cutibacterium*, and *Clostridium sensu stricto* 1, and fungi *Fusarium*, *Ruhlandiella*, *Lactariu*s, *Inocybe*, *Mortierella*, *Fusicolla*, Didymellaceae, and *Cladosporium* were keystone in gallstone (Figure 6B). Altogether, these results indicate more diverse and complex microbial interactions in gallstone than bile in the gallbladder, and the fungi and its interactions with bacteria might play a key role in the formation of gallstone.

### Potential functions of bacteria and fungi in the gallbladder of GSD

**Figure 7:**
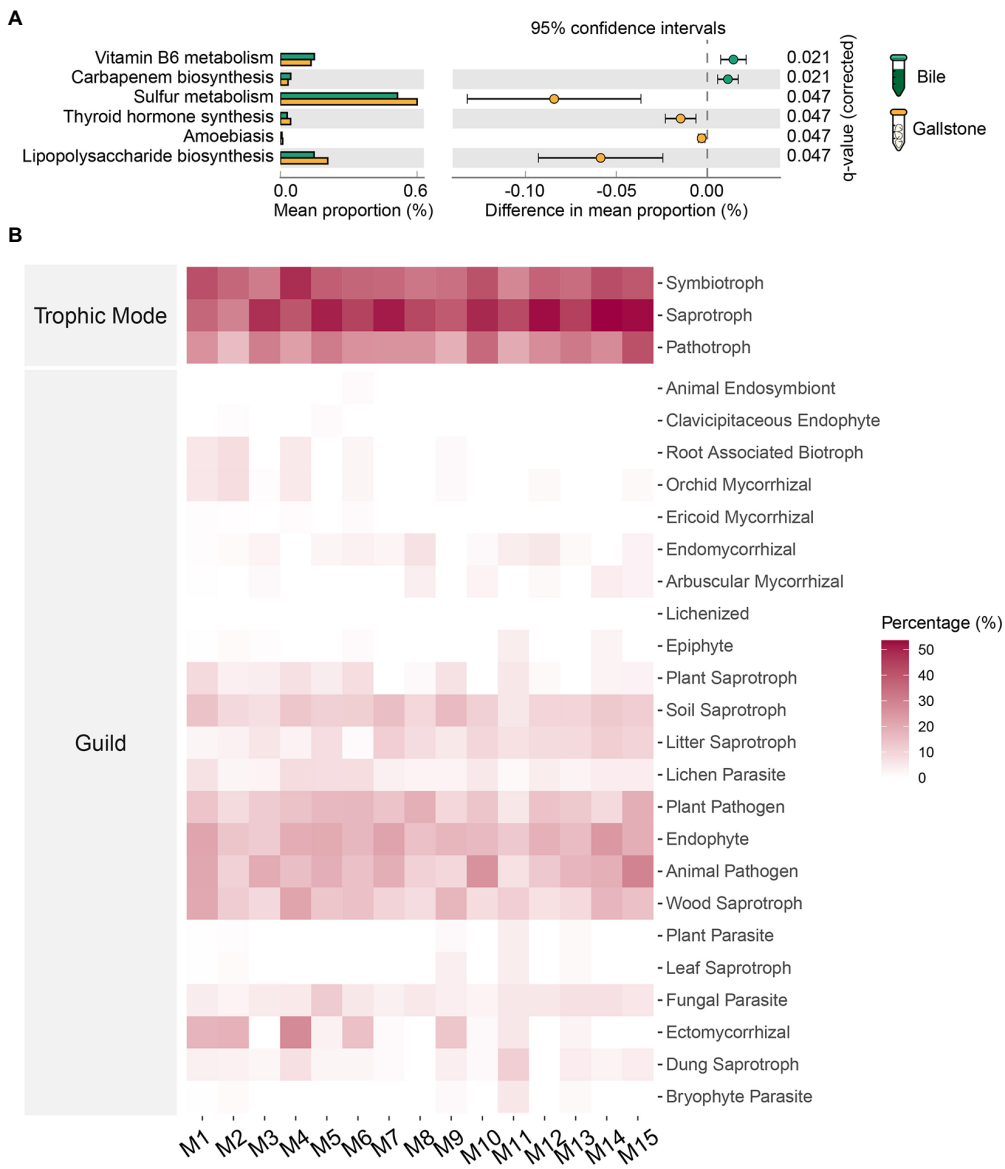
Predicted potential functions of microbiota in gallbladder of GSD patients with chronic cholecystitis. **(A)** Extended error bar of differential bacterial pathways between bile and gallstone. Statistic was performed by a two-sided *t* test (equal variance) with Storey False Discovery Rate (FDR) in STAMP. **(B)** Heatmap of fungal traits (trophic modes and guilds) in gallbladder. “M” represented the module of constructed network performed by microeco package.

To understand the potential functions of the gallbladder microbiome, we further compared the predicted outcomes of bacteria between bile and gallstone (Figure 7A) and analyzed the fungal trophic mode and guild in the gallbladder (Figure 7B). Vitamin B6 metabolism and carbapenem biosynthesis of bacteria were higher in bile than gallstone (Figure 7A). Conversely, bacterial sulfur metabolism, lipopolysaccharide biosynthesis, thyroid hormone synthesis, and amoebiasis were significantly enriched in gallstones (Figure 7A).

For fungi, we used the FUNGuild database to identify species guilds (Figure 7B). Saprotroph was the most abundant trophic mode, followed by modes symbiotroph and pathotroph. Endophyte and animal pathogen were the two dominant guilds. Additionally, fungal parasite, ectomycorrhizal, wood saprotroph, plant pathogen, lichen parasite, litter saprotroph, and soil saprotroph were also observed in the gallbladder mycobiome. Collectively, these results demonstrated that bacterial sulfur metabolism and fungal saprotroph and animal pathogen in the gallbladder might contribute to the formation of gallstones.

## Discussion

Increasing evidence has demonstrated the importance of microbiota in the development of many diseases. GSD is a major type of gallbladder disease, which affects 10–20% of the global adult population (Grigor'eva and Romanova, 2020). Multikingdom gallbladder microbiome analysis by metagenomic sequencing provides a new opportunity to explore the gallbladder mycobiome landscape in GSD. Accordingly, we used metagenomic sequencing to show for the first time that the gallbladder gallstone mycobiome in GSD subjects is similar to paired bile mycobiome, characterized by the same diversity and richness and a few differential gallstone-related fungal species. However, the interkingdom correlations between the mycobiome and bacteriome and the intrakingdom correlations within them were marked as more gallstone-related correlations. In addition, we first found that archaea (such as DSEG) were significantly thriving in bile, indicating the association thereof with gallbladder microbiome dysbiosis in GSD. These data fill an important gap in our current understanding of the potential role of gallbladder microbiome profile in GSD.

Research on the bile and gallstone microbiota and their role in biliary disease is quite rare. To our knowledge, no previous studies have described changes in the diversity of the human gallbladder mycobiome in GSD, although the fungus *Candida* in bile was recently reported by Serra et al. based on the culture method (Serra et al., 2021). To identify consistent gallbladder fungal signatures for bile and gallstone, we first combined both sex and types of gallstones to generally observe the changes. Our data showed a significant difference in evenness between bile and gallstone but not in the Shannon diversity and richness. One explanation could be that the species distribution was more uniform in gallstone than in bile (Supplementary Figure S9), which may have led to a more significant difference in the evenness of the samples. In addition, our PCoA results also suggest different trends of mycobiome between bile and gallstone samples. However, the pseudo-*F* values obtained in the PCoA remained low (1.81), indicating that larger unknown factors may contribute to variations between the subjects.

In this exploratory study to identify more potential taxa that might be associated with bile and gallstone, we included fungal species with a relative abundance of >0.005%. As a result, we found markedly differential fungal species, including taxa with lower abundance in gallstone samples. To date, limited information is available about the role of these differential fungal species in GSD, especially for the taxa with very low abundance (such as *C. sublineola*). Further functional research on the fungal markers might help understand the role of the mycobiome in gallbladder diseases.

Ascomycota and Basidiomycota constitute the major phyla of the kingdom fungi in the gallbladder. It was previously revealed that the Ascomycota:Basidiomycota ratio in the gut could represent a fungal dysbiosis index to differentiate diseases (Sokol et al., 2017; Cao et al., 2021). We found that it was higher in gallstone than in bile, implying a dysbiosis in bile, thus leading to stone formation in the gallbladder. Genera *Aspergillus* and *Candida* are two of the predominant members in the gallbladder mycobiome, some of which are widely reported to be pathogenic to humans and animals. One reason for the phenomena might be that members of *Aspergillus* can survive in both highly acidic and mildly basic conditions and at a wide range of temperatures in addition to producing secondary metabolites mycotoxins that are capable of causing disease and death in humans (Bennett and Klich, 2003). Importantly, *Candida* spp. are commonly associated with humans. The genus *Candida* comprises over 10 species that are known to induce opportunistic infections in humans, especially *C. albicans* (Yapar, 2014; Kapitan et al., 2019). In line with that, *C. albicans* was the most abundant species in both bile and gallstone.

Numerous studies have identified bile bacterial alterations in biliary diseases, but there is limited evidence to compare gallbladder microbiome differences between bile and gallstone. Our results are consistent with those of previous studies, demonstrating that phyla, Firmicutes, Bacteroidetes, Actinobacteria, and Proteobacteria, predominate in the bile bacteriome (Shen et al., 2015; Molinero et al., 2019). We found that GSD subjects were associated with significant alterations in bacterial composition between bile and gallstone samples. Notably, kingdom bacteria was significantly enriched in gallstone, demonstrating that bacteria could be a key factor contributing to stone formation. Proteobacteria which include many pathogenic species was enriched in gallstone, implying its potential role contributing to the formation of stone (Timur Liwinski et al., 2019). A recent study demonstrated that bile acid and cholesterol metabolism dysfunction contributed to cholesterol gallstone formation (Hu et al., 2022). We observed a significant increase in the cholesterol-reducing anaerobic genus *E. coprostanoligenes* in gallstone of patients with GSD (Ren et al., 1996; Le et al., 2022). One possible explanation could be that the gallstones obtained in this study were mostly cholesterol stones (85.3%). In addition, *B. plebeius*, which was found in the human gut and could become an opportunistic pathogen if it escapes the gut, was thriving in gallstone.^3^ *Clostridium sensu stricto* 1, referring to the *Clostridium* cluster I, which has a potential causative capacity, was associated with gallstone (Lopetuso et al., 2013). In addition, *Clostridiales* which can produce trimethylamine (TMA), the precursor of trimethylamine N-oxide (TMAO), was a biomarker in gallstone (Wang et al., 2015). Consistent with this, Chen Y. et al. (2019) found that high TMAO levels were positively associated with the presence of gallstone disease in humans. These results indicate that *Clostridiales* may contribute to GSD by a mechanism based on its metabolite TMA/TMAO signaling.

Archaea are widespread microorganisms that live in a variety of natural and host-associated ecosystems (Baker et al., 2020). Several aquatic microbiota types such as DSEG and Rhodobacteraceae were dominant in bile, which might be because of bile’s liquid environment, indicating that the archaea are a major group that thrives in extreme environments. However, it should be noted that we assigned the Archaea according to data of 16S rRNA genes for bacteria and not the archaea-specific genes in this study. Further research on the archaeal markers and functions might help uncover the role of the archaeome in gallbladder diseases.

It should be noted that understanding the microbial interactions are of particular significance in human health and disease. Indeed, both positive and negative connections between bacteria and fungi in the gut have been reported (Richard and Sokol, 2019). Thus, we analyzed the bacterial and fungal interkingdom and intrakingdom interactions in gallbladder. In general, our data indicate a complex microbial ecologic community in gallstones and further demonstrate that the unique environment of gallstones promotes microbial interactions; in turn, the complex interactions may boost the formation and growth of gallstones in the gallbladder.

In addition, we found a reconstruction of microbial co-occurrence network in gallstone. First, the number of nodes increased in the network of gallstones. Second, the key modules changed within a network of gallstones. Third, the relationships altered between the taxa, and even in the same two taxa, such as the *Muribaculum* and *Rothia* (positive in bile, but negative in gallstone; Figures 5A,B; Supplementary Tables S3, S4). Interestingly, the ratio of positive and negative correlations seems to maintain a “relative balance” (positive/negative = 1.3) in the bacteria–bacteria network in both bile and gallstone. The ratio is close to 2 (1.8) in the networks of fungi–fungi and bacteria–fungi in bile, whereas it is close to 1 in these networks in gallstone. This suggests that (1) microbial interactions tend to affect homeostasis in the gallbladder, and that (2) fungal disorder in bile might play a vital role in GSD.

Importantly, in this study, we found that the bacterial communities are largely different, while the fungal colonies are similar between bile and gallstone in GSD. This interesting phenomenon could be explained by three points. First, the bacteria were indeed the most major members of microorganisms in bile while fungi were a smaller group than bacteria. Moreover, we detected a group of archaea accounting for a large proportion in bile which hardly were detected in gallstone in bacterial analysis (such as DSEG, Figure 2). Second, the number of negative interactions (e.g., resource competition) of bacteria–bacteria network was more than those of fungi–fungi in bile and gallstone (Table 2). Therefore, this might cause a large change of bacteria not fungi. Last, there are distinct types of environments between bile and gallstone—liquid for bile whereas solid for gallstone, as well as disparate environmental factors, mainly water, bile acids/salts, and inorganic ions for bile but cholesterol, calcium salts, and bilirubin for gallstone. Thus, there are more room and resource of bile than gallstone to survive for microbes so that bacteria altered largely for surviving. Altogether, it is implied that most fungi and a small part bacteria detected in current study might contribute to the formation of gallstone.

Hu et al. identified the gut bacterial *Desulfovibrionales* as a key taxon contributing to cholesterol gallstone formation (Hu et al., 2022). Furthermore, *Desulfovibrionales* are responsible for metabolizing dietary and host-derived sulfur-containing compounds (Peck et al., 2019). In line with this, bacterial sulfur metabolism was significantly active in gallstone according to function prediction analysis in this study. Lipopolysaccharide (LPS) is an endotoxin derived from the outer membrane of Gram-negative bacteria. LPS can cause many health problems, particularly in people who have thyroid and autoimmune thyroid disorders. Indeed, we found high thyroid hormone synthesis activity of bacteria in gallstones. In addition, LPS can cause an acute inflammatory response by triggering the release of a great number of inflammatory cytokines (Lopes, 2016). One possible interpretation is that the subjects recruited in this study have chronic cholecystitis complications.

Notably, this study had some limitations. First, because of ethical and social issues, we could not obtain healthy bile samples as the control group. Accordingly, collecting bile samples from the gallbladders of subjects without hepatobiliary disease from liver transplant donors for the control or reference bile microbiota group may be an alternative method (Molinero et al., 2019).

Second, we used the marker gene (16S/ITS) amplicon sequencing technology for microbial analysis, which did not provide the functional information of microbiota in the gallbladder. Meanwhile, we used the reference database approach for bacterial and fungal reads classification, which may allow the loss of a considerable part of novel microbiota owing to the absence of a reference data. As such, shotgun metagenomic sequencing is an alternative solution. In addition, an archaea-specific analysis method is further required.

Third, the gut microbiome community is also likely to be linked with the formation of gallstones (Hu et al., 2022; Zhao et al., 2022). Our study did not excavate the spectrum of the gut microbiome. Thus, the location difference of gut not only the feces need to be further explored. Moreover, the microbial metabolites (such as secondary bile acids) are now suggested to be very important in health and disease (de Vos et al., 2022; Hu et al., 2022). Metabonomics may be a useful approach to uncover the GSD-related microbial metabolites in the future.

Fourth, because we did not include patients with longitudinal follow-up and prognosis, we were unable to study the possibility of preventing GSD by evaluating the risk of GSD before its progression. Future studies should recruit individuals with prognoses to validate the value of predicting full-blown GSD by gallbladder microbial profiles.

Lastly, other external factors, including diet and lifestyle changes—which are known to affect the microbiome (David et al., 2014; Strasser et al., 2021)—were not assessed in this study. Nonetheless, these findings provided new insights into our understanding of the role of bacteriome and mycobiome alterations in bile and gallstone. Further larger-scale studies are needed to explore the confounding factors and cause–effect between microbiome and GSD.

## Conclusion

This study demonstrated that large and complex bacteria and fungi inhabited in the gallbladder of patients with GSD, and gallstone was characterized by significantly altered bacterial taxonomic composition. In addition, the bacterial**–**bacterial, fungal**–**fungal and bacterial**–**fungal correlations were strengthened compared to bile in the gallbladder of patients with GSD. As such, mycobiome, bacteriome, and even archaeome and their interactions might contribute to the formation of gallstone. We believe that our first paired bile**–**gallstone microbiome study has taken an important step in this field.

## Data availability statement

The data presented in the study are deposited in the Genome Sequence Archive (https://ngdc.cncb.ac.cn/gsa) repository in the National Genomics Data Center (NGDC), accession numbers CRA008937 and CRA008920.

## Ethics statement

The studies involving human participants were reviewed and approved by the Institutional Ethics Review Board of the Third People’s Hospital of Chengdu. The patients/participants provided their written informed consent to participate in this study.

## Author contributions

YL, TZ, and JH designed the study. YJL and QY obtained the grants. JH analyzed the data and obtained plots. JT, XZ, KY, AZ, and YL enrolled the patients and participated in the clinical part of the study. JH and TZ wrote the draft of the manuscript. All the authors participated in the discussion and reviewed the manuscript.

## Funding

This work was supported by the National Natural Science Foundation of China (82170887), the Chengdu High-level Key Clinical Specialty Construction Project, and the Science and Technology Project of The Health Planning Committee of Sichuan Municipality (20PJ211).

## Conflict of interest

The authors declare that the research was conducted in the absence of any commercial or financial relationships that could be construed as a potential conflict of interest.

## Publisher’s note

All claims expressed in this article are solely those of the authors and do not necessarily represent those of their affiliated organizations, or those of the publisher, the editors and the reviewers. Any product that may be evaluated in this article, or claim that may be made by its manufacturer, is not guaranteed or endorsed by the publisher.

## Footnotes

^1^ https://github.com/ChiLiubio/file2meco

^2^ https://beikolab.cs.dal.ca/software/STAMP

^3^ https://www.ncbi.nlm.nih.gov/genome/?term=txid310297
