# Supplementary material for: Landscape in the gallbladder mycobiome and bacteriome of patients undergoing cholelithiasis with chronic cholecystitis
Source: Front Microbiol. 2023 Mar 22;14:1131694. doi: 10.3389/fmicb.2023.1131694 (PMC10073429; doi:10.3389/fmicb.2023.1131694)
Supplement: Supplementary file 1 [file Data_Sheet_1.docx]

Supplementary Materials

# Supplementary Figures


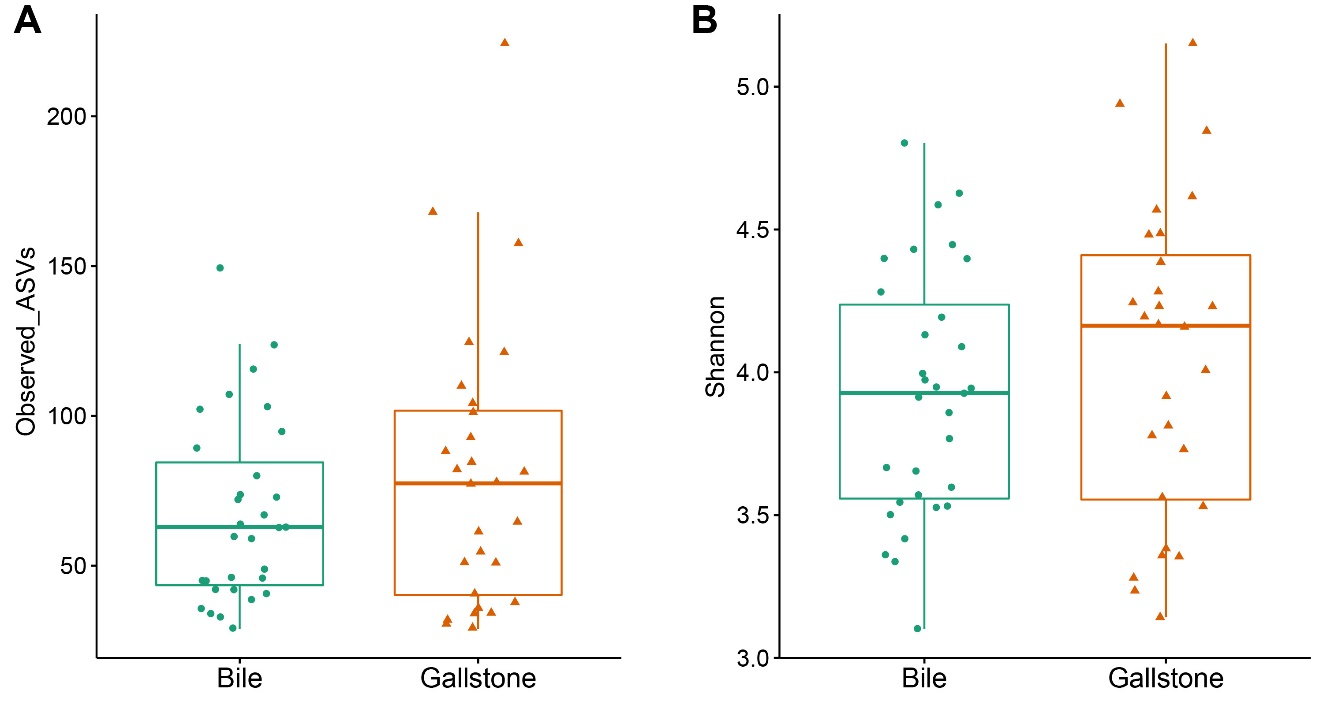


**Fig. S1. Bacterial diversity** **between bile and gallstone from GSD patients with chronic cholecystitis.** Boxplot showed the bacterial (**A**) Observed ASVs and (**B**) Shannon diversity between bile (n = 31) and gallstone (n = 28). Statistical significance was performed by a Kruskal-Wallis (pairwise) test. There were no significant differences between bile and gallstone samples in Observed (*P* = 0.45) and Shannon diversity (*P* = 0.36).


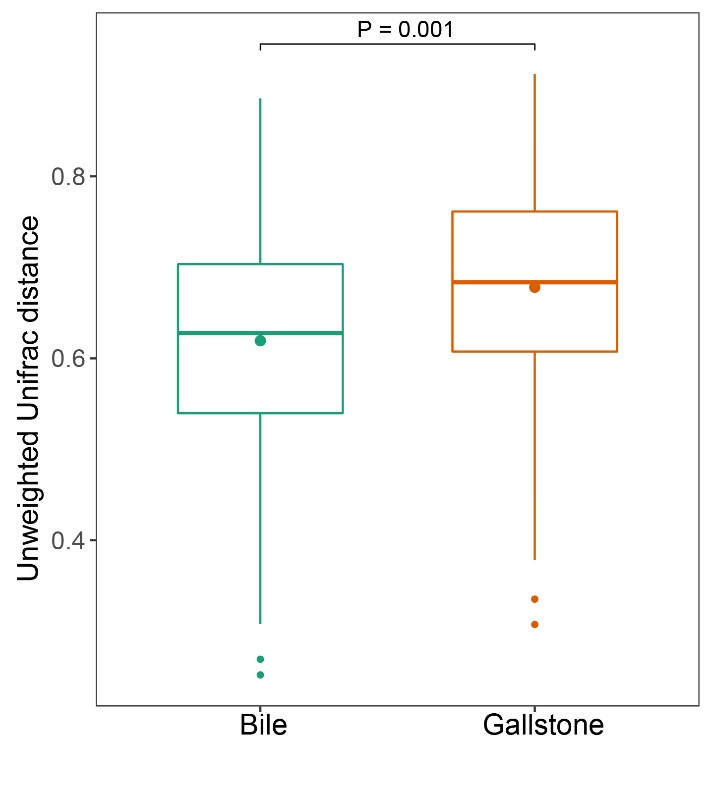


**Fig. S2.** **Boxplot of bacterial Unweighted Unifrac distances between bile (n = 31) and gallstone (n = 28) from GSD patients with chronic cholecystitis.** Statistical significance was performed by a pairwise PERMANOVA test (999 permutations).


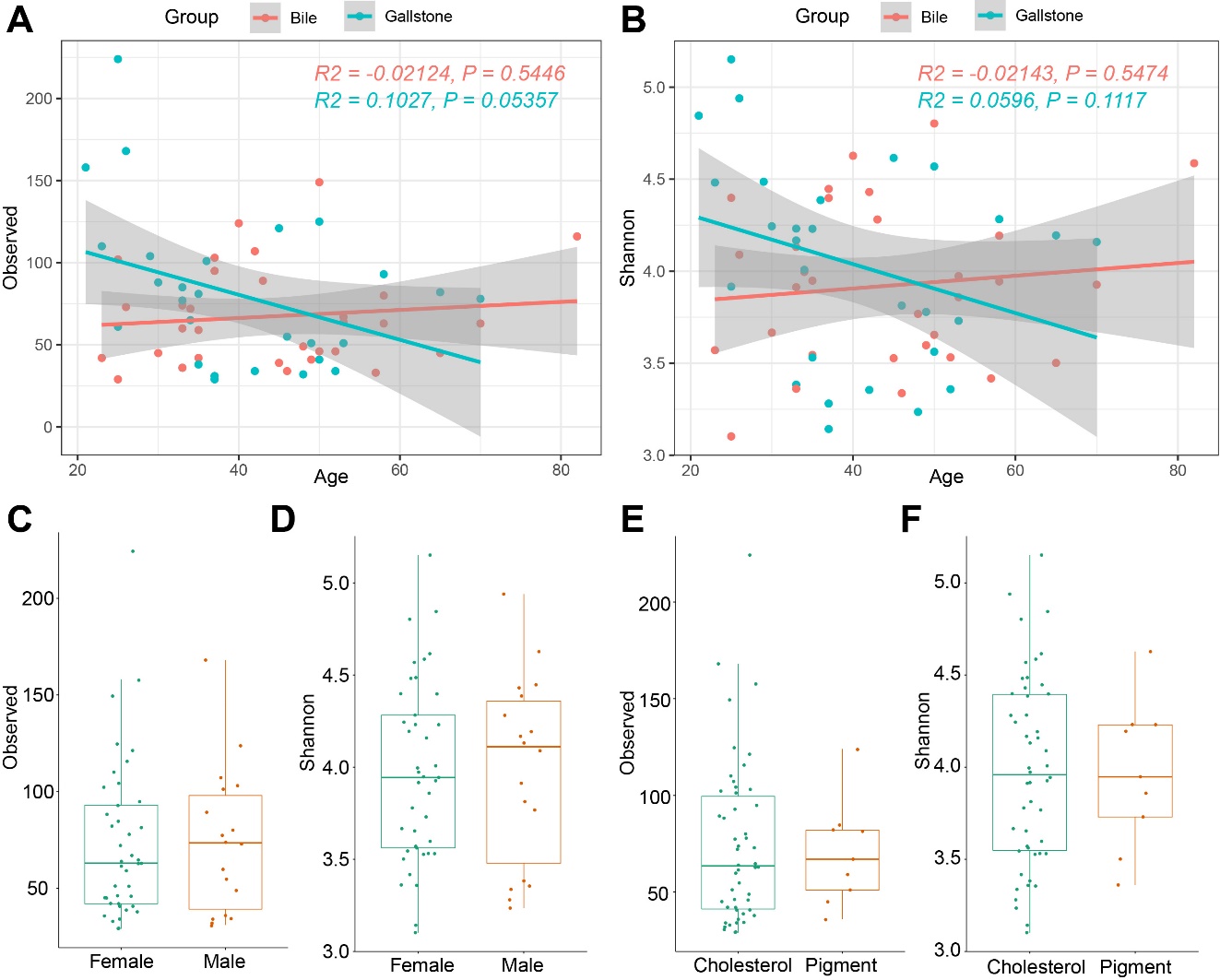


**Fig. S3.** **No significant association was found between gallbladder bacterial alpha diversity, age, sex and stone type.** Observed ASVs (**A**) and Shannon diversity (**B**) correlate with age in bile samples and gallstone samples. Statistical significance was determined by linear regression (*F*-statistic). The *R2* was an adjusted R-squared. Bacterial Observed ASVs (**C**) and Shannon diversity (**D**) between men (n = 18) and women (n = 41) in GSD subjects. Statistical significance was determined by Kruskal-Wallis (pairwise) test. There were no significant differences between women and men in Observed (*P* = 0.97) and Shannon diversity (*P* = 0.99). Bacterial Observed ASVs (**E**) and Shannon diversity (**F**) between cholesterol (n = 50) and pigment (n = 9) stone in GSD subjects. Statistical significance was determined by Kruskal-Wallis (pairwise) test. There were no significant differences between cholesterol and pigment stone in Observed (*P* = 0.83) and Shannon diversity (*P* = 0.97).


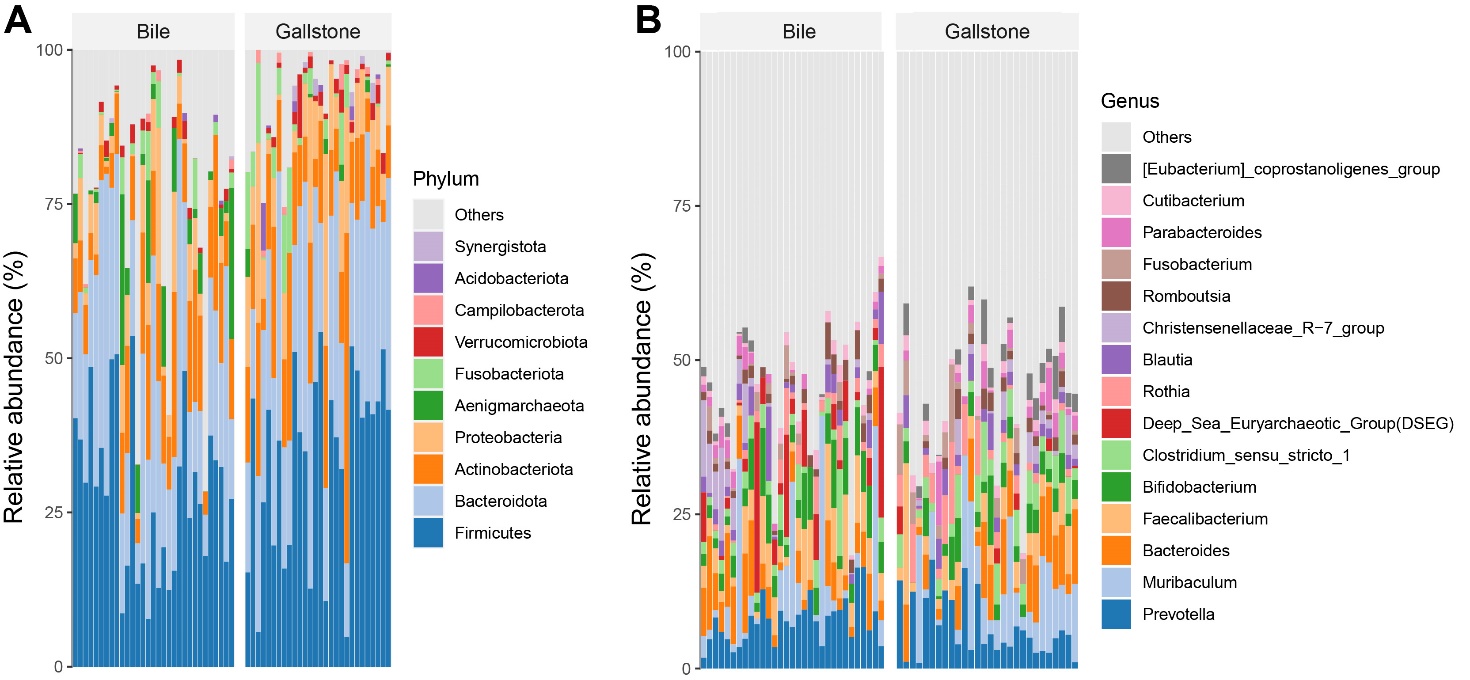


**Fig. S4. Taxonomic distribution of gallbladder bacteriome in bile and gallstone samples**. Taxonomic distribution of bacterial taxa is shown at the (**A**) phylum and (**B**) genus level between bile and gallstone samples. Only the top 10 (phyla) or 15 (genera) taxa were presented in the bar plot according to individual relative abundance.


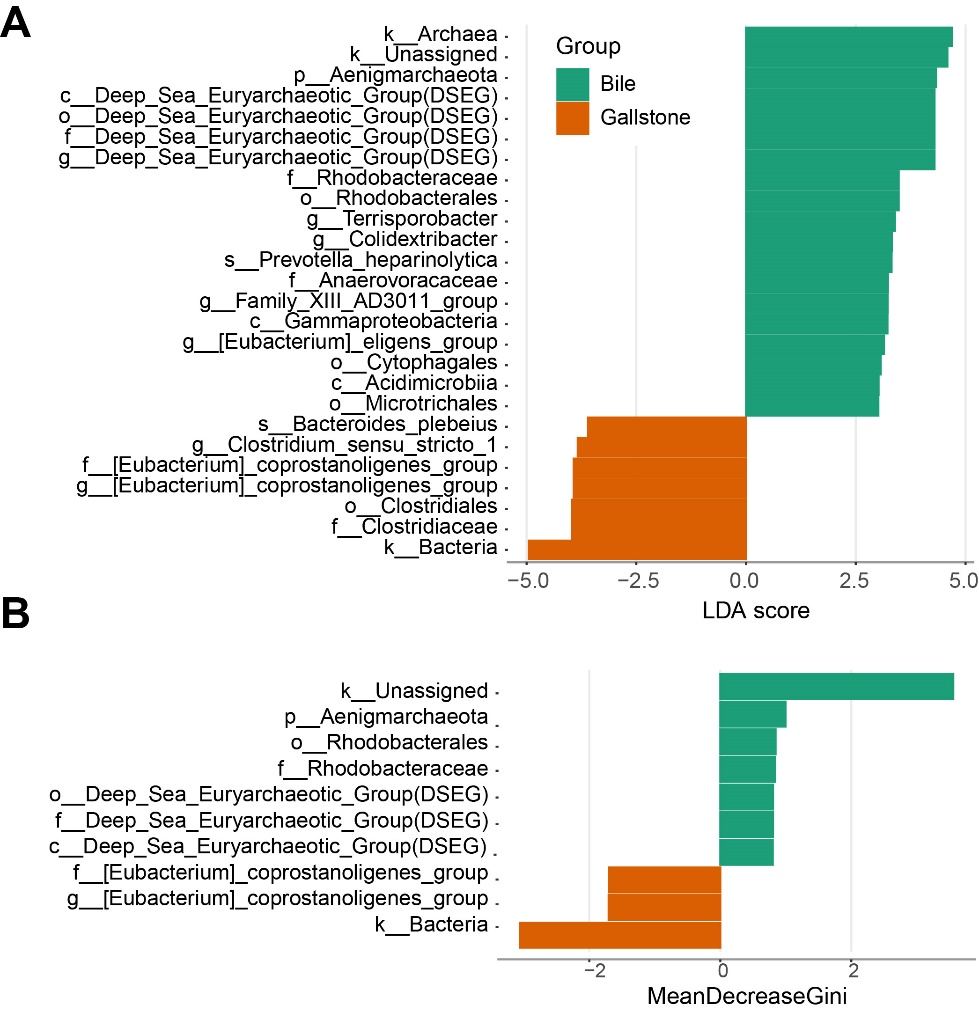


**Fig. S5. Bacterial biomarkers between bile and gallstone samples.** (**A**) LEfSe barplot showed the different abundance of bacteria between bile and gallstone samples (LDA score > 3.0). (**B**) Random forest (RF) depicted the biomarkers of bile and gallstone samples.


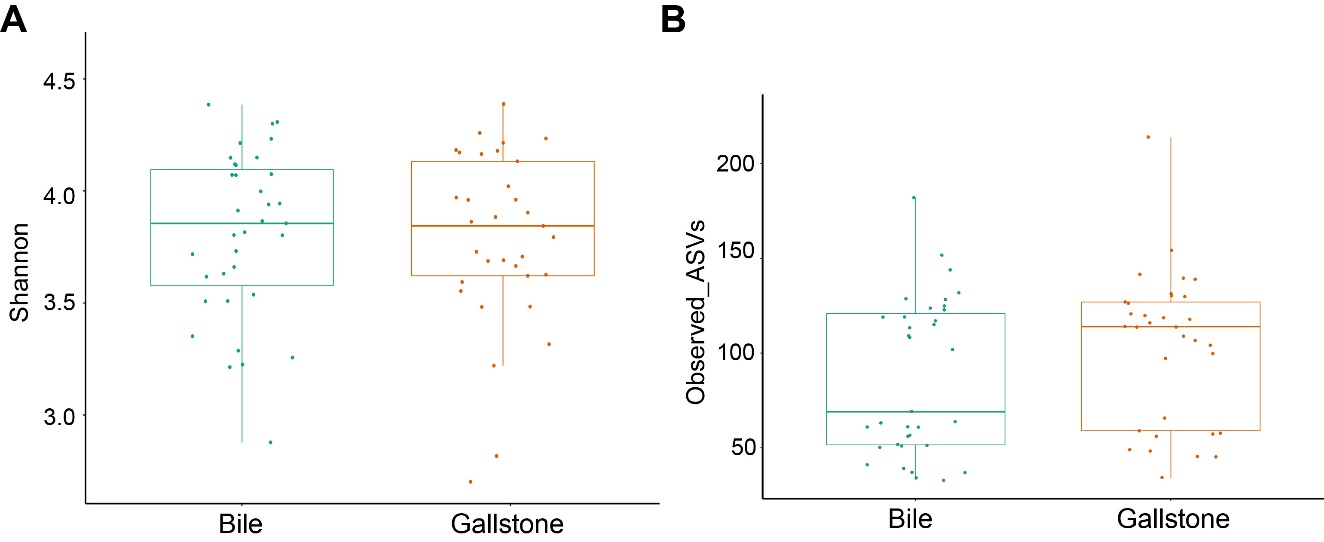


**Fig. S6. Fungal diversity between bile and gallstone from GSD patients with chronic cholecystitis.** Boxplot showed the bacterial (**A**) Shannon diversity and (**B**) Observed ASVs between bile (n = 35) and gallstone (n = 33). Statistical significance was performed by a Kruskal-Wallis (pairwise) test. There were no significant differences between bile and gallstone samples in Observed (*P* = 0.18) and Shannon diversity (*P* = 0.88).


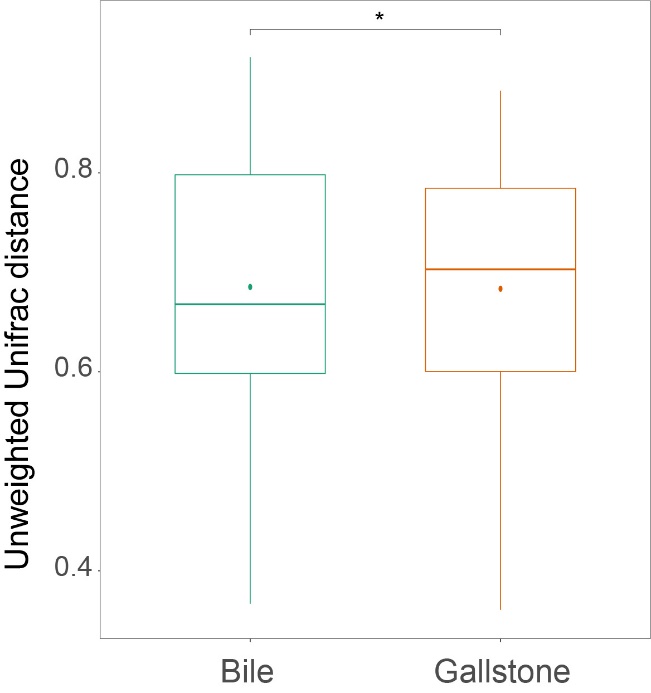


**Fig. S7. Boxplot of bacterial Unweighted Unifrac distances between bile (n = 35) and gallstone (n = 33) from GSD patients with chronic cholecystitis.** Statistical significance was performed by a pairwise PERMANOVA test (999 permutations). * *P* (= 0.038) < 0.05.


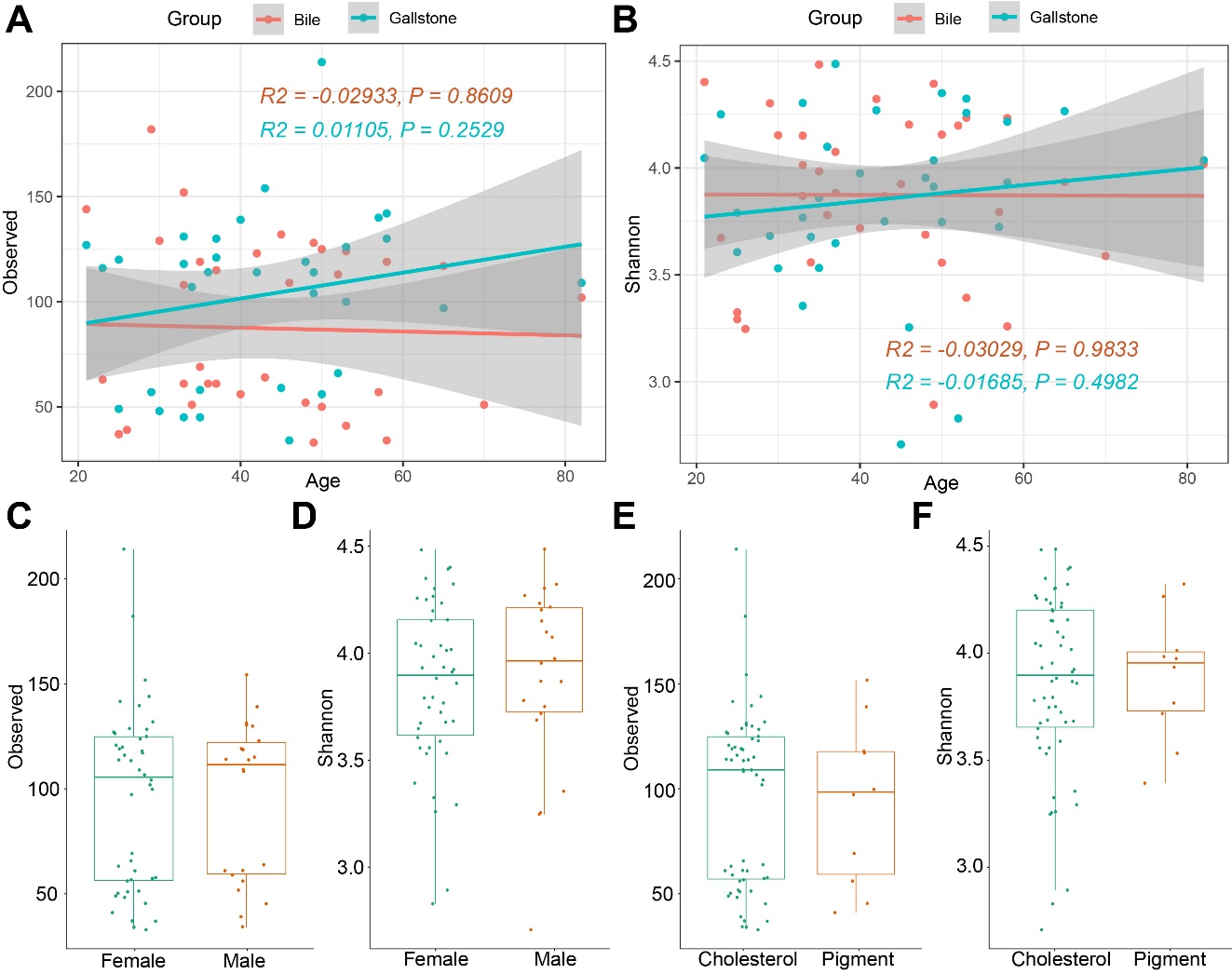


**Fig. S8. No significant association was found between gallbladder fungal alpha diversity, age, sex and stone type.** Observed ASVs (**A**) and Shannon diversity (**B**) correlate with age in bile samples and gallstone samples. Statistical significance was determined by linear regression (*F*-statistic). The *R2* was an adjusted R-squared. Fungal Observed ASVs (**C**) and Shannon diversity (**D**) between men (n = 22) and women (n = 46) in GSD subjects. Statistical significance was determined by Kruskal-Wallis (pairwise) test. There were no significant differences between women and men in Observed (*P* = 0.87) and Shannon diversity (*P* = 0.53). Fungal Observed ASVs (**E**) and Shannon diversity (**F**) between cholesterol (n = 58) and pigment (n = 10) stone in GSD subjects. Statistical significance was determined by Kruskal-Wallis (pairwise) test. There were no significant differences between cholesterol and pigment stone in Observed (*P* = 0.83) and Shannon diversity (*P* = 0.97).


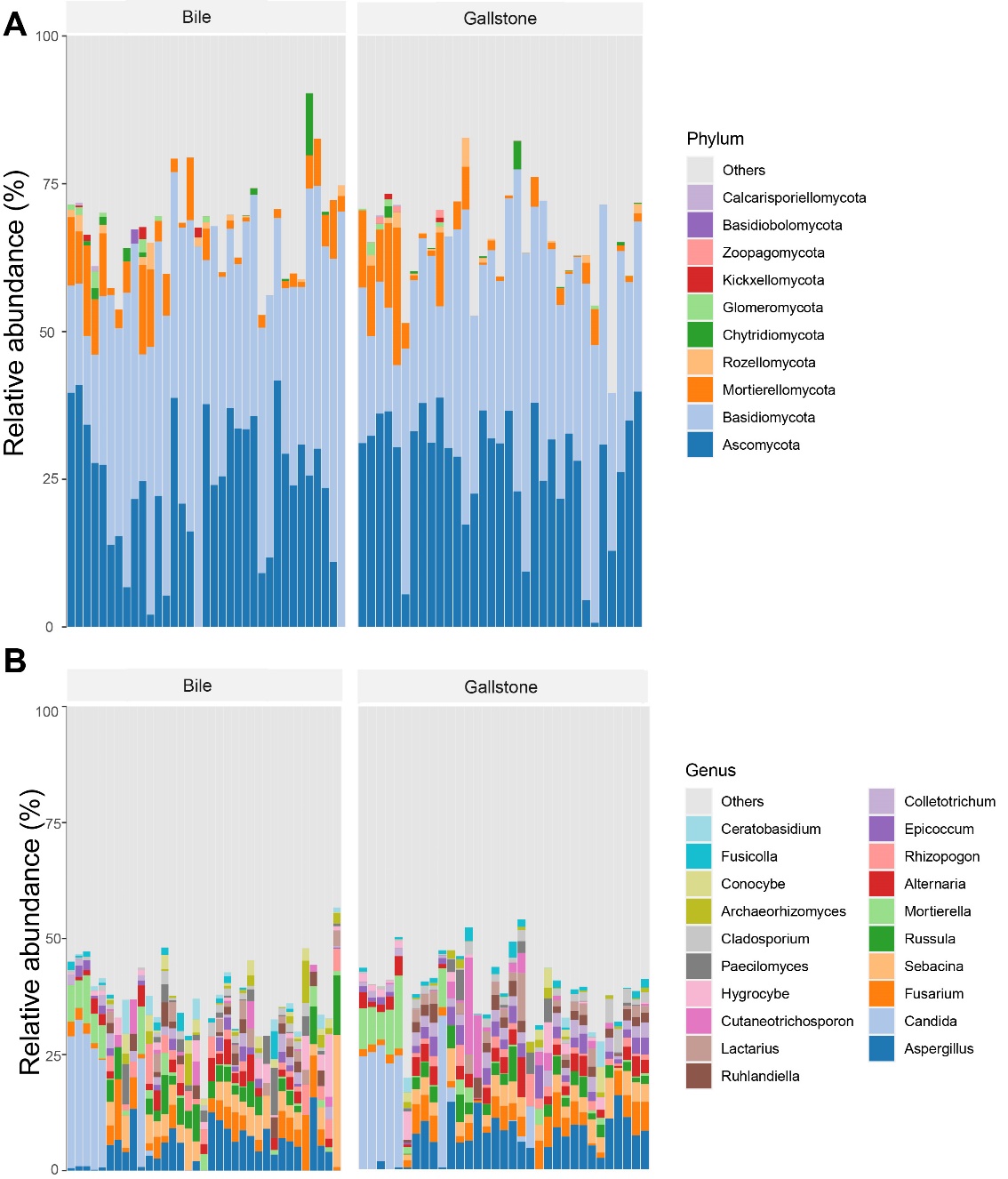


**Fig. S9. Taxonomic distribution of gallbladder mycobiome in bile and gallstone samples**. Taxonomic distribution of fungal taxa is shown at the (**A**) phylum and (**B**) genus level between bile and gallstone samples. Only the top 10 (phyla) or 20 (genera) taxa were presented in the bar plot according to individual relative abundance.


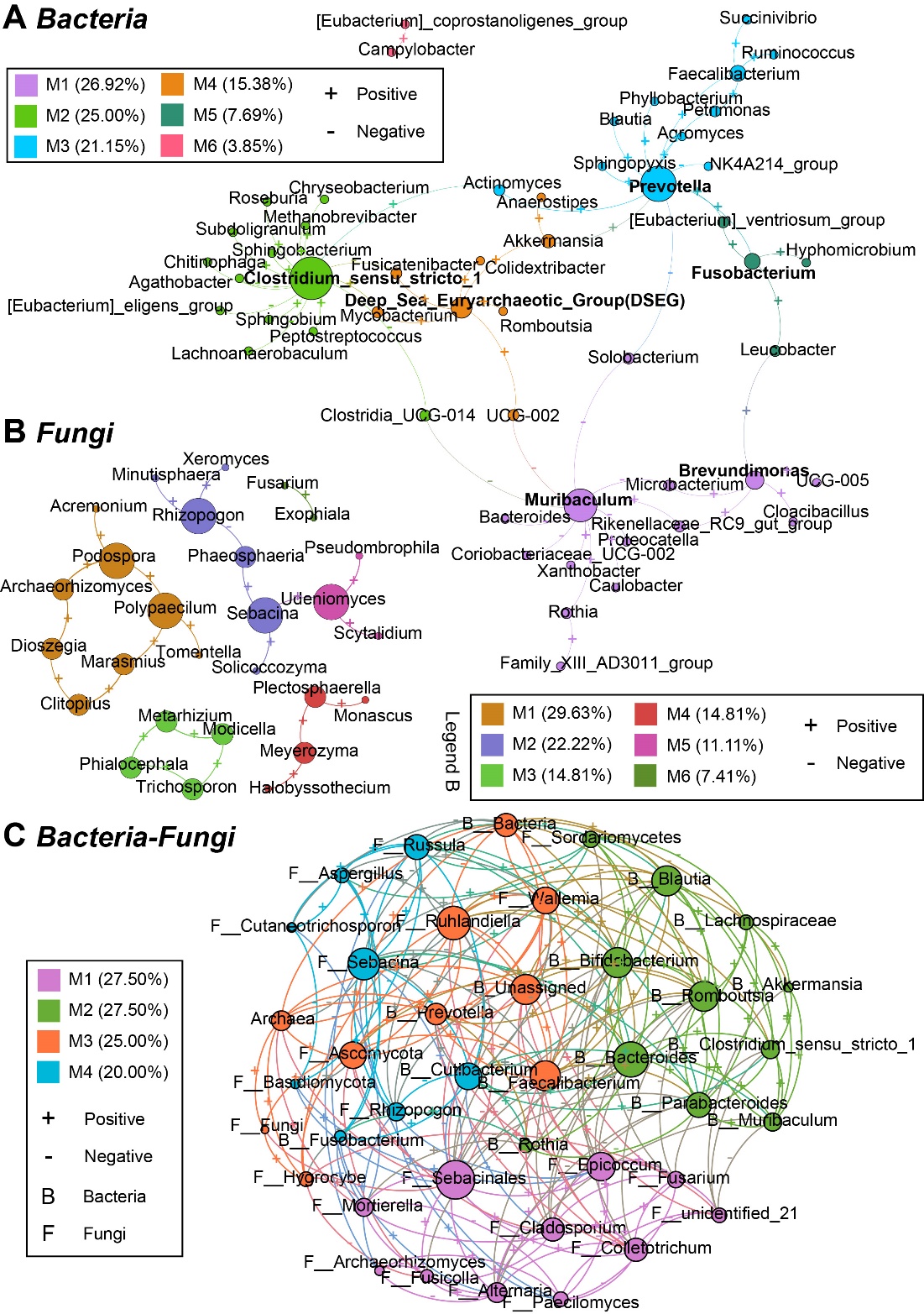


**Fig. S10. Global Co-occurrence networks in gallbladder of GSD patients with chronic cholecystitis.** **A–C** Bacterial–bacterial (**A**), fungal–fungal (**B**) and bacterial–fungal (**C**) co-occurrence networks of bile and gallstone samples at the genus level. Node size was presented by its degree (3–15). “M” represented the module of the network.

# Supplementary Tables

**Table S1 General information on subjects in this study**

| **Subjects** | **Disease** | **Sample-type** | **Age** | **Sex** | **Types-of-gallstone** | **Data** |
| --- | --- | --- | --- | --- | --- | --- |
| Subject1 | Cholelithiasis with chronic cholecystitis | Bile | 33 | Male | Cholesterol | 16S ＆ ITS |
|  |  | Gallstone |  |  |  | 16S ＆ ITS |
| Subject2 | Cholelithiasis with chronic cholecystitis | Bile | 37 | Male | Cholesterol | 16S ＆ ITS |
|  |  | Gallstone |  |  |  | 16S ＆ ITS |
| Subject3 | Cholelithiasis with chronic cholecystitis | Bile | 58 | Male | Cholesterol | 16S ＆ ITS |
|  |  | Gallstone |  |  |  | 16S ＆ ITS |
| Subject4 | Cholelithiasis with chronic cholecystitis | Bile | 53 | Female | Cholesterol | 16S ＆ ITS |
|  |  | Gallstone |  |  |  | 16S ＆ ITS |
| Subject5 | Cholelithiasis with chronic cholecystitis | Bile | 42 | Male | Cholesterol | 16S ＆ ITS |
|  |  | Gallstone |  |  |  | 16S ＆ ITS |
| Subject6 | Cholelithiasis with chronic cholecystitis | Bile | 82 | Female | Cholesterol | 16S ＆ ITS |
|  |  | Gallstone |  |  |  | 16S ＆ ITS |
| Subject7 | Cholelithiasis with chronic cholecystitis | Bile | 25 | Female | Cholesterol | 16S ＆ ITS |
|  |  | Gallstone |  |  |  | 16S ＆ ITS |
| Subject8 | Cholelithiasis with chronic cholecystitis | Bile | 43 | Male | Cholesterol | 16S ＆ ITS |
|  |  | Gallstone |  |  |  | 16S ＆ ITS |
| Subject9 | Cholelithiasis with chronic cholecystitis | Bile | 40 | Male | Pigment | 16S ＆ ITS |
|  |  | Gallstone |  |  |  | 16S ＆ ITS |
| Subject10 | Cholelithiasis with chronic cholecystitis | Bile | 35 | Female | Pigment | 16S ＆ ITS |
|  |  | Gallstone |  |  |  | 16S ＆ ITS |
| Subject11 | Cholelithiasis with chronic cholecystitis | Bile | 35 | Female | Cholesterol | 16S ＆ ITS |
|  |  | Gallstone |  |  |  | 16S ＆ ITS |
| Subject12 | Cholelithiasis with chronic cholecystitis | Bile | 33 | Male | Cholesterol | 16S ＆ ITS |
|  |  | Gallstone |  |  |  | 16S ＆ ITS |
| Subject13 | Cholelithiasis with chronic cholecystitis | Bile | 52 | Female | Cholesterol | 16S ＆ ITS |
|  |  | Gallstone |  |  |  | 16S ＆ ITS |
| Subject14 | Cholelithiasis with chronic cholecystitis | Bile | 48 | Male | Cholesterol | 16S ＆ ITS |
|  |  | Gallstone |  |  |  | 16S ＆ ITS |
| Subject15 | Cholelithiasis with chronic cholecystitis | Bile | 58 | Female | Cholesterol | 16S ＆ ITS |
|  |  | Gallstone |  |  |  | 16S ＆ ITS |
| Subject16 | Cholelithiasis with chronic cholecystitis | Bile | 37 | Female | Cholesterol | 16S ＆ ITS |
|  |  | Gallstone |  |  |  | 16S ＆ ITS |
| Subject17 | Cholelithiasis with chronic cholecystitis | Bile | 23 | Female | Cholesterol | 16S ＆ ITS |
|  |  | Gallstone |  |  |  | 16S ＆ ITS |
| Subject18 | Cholelithiasis with chronic cholecystitis | Bile | 25 | Female | Cholesterol | 16S ＆ ITS |
|  |  | Gallstone |  |  |  | 16S ＆ ITS |
| Subject19 | Cholelithiasis with chronic cholecystitis | Bile | 46 | Male | Cholesterol | 16S ＆ ITS |
|  |  | Gallstone |  |  |  | 16S ＆ ITS |
| Subject20 | Cholelithiasis with chronic cholecystitis | Bile | 30 | Female | Cholesterol | 16S ＆ ITS |
|  |  | Gallstone |  |  |  | 16S ＆ ITS |
| Subject21 | Cholelithiasis with chronic cholecystitis | Bile | 50 | Female | Cholesterol | 16S ＆ ITS |
|  |  | Gallstone |  |  |  | 16S ＆ ITS |
| Subject22 | Cholelithiasis with chronic cholecystitis | Bile | 33 | Female | Pigment | 16S ＆ ITS |
|  |  | Gallstone |  |  |  | 16S ＆ ITS |
| Subject23 | Cholelithiasis with chronic cholecystitis | Bile | 50 | Female | Cholesterol | 16S ＆ ITS |
|  |  | Gallstone |  |  |  | 16S ＆ ITS |
| Subject24 | Cholelithiasis with chronic cholecystitis | Bile | 57 | Female | Cholesterol | 16S ＆ ITS |
|  |  | Gallstone |  |  |  | 16S ＆ ITS |
| Subject25 | Cholelithiasis with chronic cholecystitis | Bile | 49 | Female | Cholesterol | 16S ＆ ITS |
|  |  | Gallstone |  |  |  | 16S ＆ ITS |
| Subject26 | Cholelithiasis with chronic cholecystitis | Bile | 53 | Female | Pigment | 16S ＆ ITS |
|  |  | Gallstone |  |  |  | 16S ＆ ITS |
| Subject27 | Cholelithiasis with chronic cholecystitis | Bile | 65 | Female | Pigment | 16S ＆ ITS |
|  |  | Gallstone |  |  |  | 16S ＆ ITS |
| Subject28 | Cholelithiasis with chronic cholecystitis | Bile | 29 | Female | Cholesterol | 16S ＆ ITS |
|  |  | Gallstone |  |  |  | 16S ＆ ITS |
| Subject29 | Cholelithiasis with chronic cholecystitis | Bile | 45 | Female | Cholesterol | 16S ＆ ITS |
|  |  | Gallstone |  |  |  | 16S ＆ ITS |
| Subject30 | Cholelithiasis with chronic cholecystitis | Bile | 26 | Male | Cholesterol | 16S ＆ ITS |
|  |  | Gallstone |  |  |  | 16S only |
| Subject31 | Cholelithiasis with chronic cholecystitis | Bile | 70 | Female | Cholesterol | 16S ＆ ITS |
|  |  | Gallstone |  |  |  | 16S only |
| Subject32 | Cholelithiasis with chronic cholecystitis | Bile | 36 | Male | Cholesterol | 16S ＆ ITS |
|  |  | Gallstone |  |  |  | 16S ＆ ITS |
| Subject33 | Cholelithiasis with chronic cholecystitis | Bile | 34 | Female | Cholesterol | 16S ＆ ITS |
|  |  | Gallstone |  |  |  | 16S ＆ ITS |
| Subject34 | Cholelithiasis with chronic cholecystitis | Bile | 21 | Female | Cholesterol | 16S ＆ ITS |
|  |  | Gallstone |  |  |  | 16S ＆ ITS |
| Subject35 | Cholelithiasis with chronic cholecystitis | Bile | 49 | Female | Cholesterol | 16S ＆ ITS |
|  |  | Gallstone |  |  |  | 16S ＆ ITS |

**Table S2 SparCC correlation of bacterial–fungal network in bile and gallstone**

| **Source** | **Interaction** | **Target** | **Correlation** | ***P* value** |
| --- | --- | --- | --- | --- |
| g__unidentified_1(Sebacinales) | negative | p__Ascomycota | -0.50718 | 0 |
| g__Epicoccum | negative | g__Faecalibacterium | -0.46146 | 0 |
| g__Epicoccum | negative | g__Romboutsia | -0.43511 | 0 |
| g__unidentified_1(Sebacinales) | negative | k__Fungi | -0.41554 | 0.05 |
| g__Wallemia | negative | g__Prevotella | -0.39289 | 0.01 |
| g__unidentified_1(Sebacinales) | negative | g__Faecalibacterium | -0.38805 | 0 |
| g__Colletotrichum | negative | d_Unassigned | -0.38567 | 0 |
| g__Ruhlandiella | negative | g__Prevotella | -0.38426 | 0 |
| c__Sordariomycetes | negative | g__Parabacteroides | -0.37063 | 0.01 |
| g__unidentified_21 | negative | g__Akkermansia | -0.36826 | 0 |
| g__Ruhlandiella | negative | p__Ascomycota | -0.36461 | 0 |
| g__Cladosporium | negative | g__Bacteroides | -0.35522 | 0 |
| g__Wallemia | negative | g__Cutibacterium | -0.34793 | 0 |
| g__Fusicolla | negative | p__Ascomycota | -0.33727 | 0 |
| g__unidentified_21 | negative | g__Muribaculum | -0.33473 | 0 |
| g__Cladosporium | negative | g__Faecalibacterium | -0.33321 | 0.02 |
| g__Wallemia | negative | p__Ascomycota | -0.3303 | 0 |
| g__Wallemia | negative | g__Romboutsia | -0.32858 | 0 |
| g__Russula | negative | g__Blautia | -0.32597 | 0 |
| g__Russula | negative | d__Bacteria | -0.32521 | 0 |
| g__Colletotrichum | negative | g__unidentified_21 | -0.3234 | 0 |
| c__Sordariomycetes | negative | f__Lachnospiraceae | -0.32089 | 0 |
| g__Epicoccum | negative | g__Bifidobacterium | -0.31922 | 0 |
| g__Fusarium | negative | g__Paecilomyces | -0.31651 | 0 |
| g__unidentified_21 | negative | g__Clostridium_sensu_stricto_1 | -0.31338 | 0 |
| g__Fusarium | negative | g__Bifidobacterium | -0.313 | 0.01 |
| g__Wallemia | negative | g__Blautia | -0.31282 | 0 |
| g__Alternaria | negative | g__Muribaculum | -0.31029 | 0 |
| g__Rhizopogon | negative | g__Cutibacterium | -0.30853 | 0.01 |
| g__Ruhlandiella | negative | d__Bacteria | -0.30602 | 0 |
| g__Ruhlandiella | negative | g__Cutibacterium | -0.30574 | 0.01 |
| g__Colletotrichum | negative | g__Faecalibacterium | -0.30417 | 0.02 |
| g__Ruhlandiella | negative | g__Blautia | -0.30341 | 0 |
| g__Cladosporium | negative | p__Ascomycota | -0.30198 | 0 |
| g__unidentified_1(Sebacinales) | negative | d_Unassigned | -0.29812 | 0 |
| g__Aspergillus | negative | g__Fusobacterium | -0.29807 | 0.02 |
| c__Sordariomycetes | negative | g__Bacteroides | -0.29572 | 0 |
| g__Colletotrichum | negative | g__Romboutsia | -0.29459 | 0 |
| g__unidentified_1(Sebacinales) | negative | g__Bifidobacterium | -0.29344 | 0.01 |
| g__Sebacina | negative | g__Prevotella | -0.29218 | 0 |
| g__Wallemia | negative | d_Unassigned | -0.29167 | 0.03 |
| g__Cutaneotrichosporon | negative | p__Basidiomycota | -0.29107 | 0.01 |
| g__Bacteroides | negative | g__Rothia | -0.29057 | 0.01 |
| g__unidentified_1(Sebacinales) | negative | g__Bacteroides | -0.28874 | 0 |
| g__Epicoccum | negative | g__Bacteroides | -0.2846 | 0.02 |
| g__Epicoccum | negative | d_Unassigned | -0.28374 | 0 |
| g__Hygrocybe | negative | g__Ruhlandiella | -0.27934 | 0.01 |
| g__Alternaria | negative | g__Parabacteroides | -0.27796 | 0 |
| g__Fusicolla | negative | g__Hygrocybe | -0.27548 | 0.01 |
| g__Russula | negative | g__Cutibacterium | -0.27535 | 0 |
| g__Epicoccum | negative | g__unidentified_21 | -0.27457 | 0 |
| g__unidentified_1(Sebacinales) | negative | g__Romboutsia | -0.27045 | 0.02 |
| g__Ruhlandiella | negative | g__Faecalibacterium | -0.26968 | 0 |
| g__Ruhlandiella | negative | d__Archaea | -0.26819 | 0.03 |
| g__Cladosporium | negative | g__Bifidobacterium | -0.26797 | 0 |
| g__Alternaria | negative | g__Bacteroides | -0.26786 | 0 |
| g__Fusarium | negative | g__Romboutsia | -0.26514 | 0.03 |
| g__Archaeorhizomyces | negative | g__Fusicolla | -0.26431 | 0.02 |
| g__Cutaneotrichosporon | negative | g__Mortierella | -0.26323 | 0.04 |
| g__Ruhlandiella | negative | d_Unassigned | -0.26266 | 0.01 |
| g__Sebacina | negative | g__Fusobacterium | -0.26171 | 0.02 |
| g__unidentified_1(Sebacinales) | negative | g__Cutibacterium | -0.26041 | 0.02 |
| g__Russula | negative | g__Parabacteroides | -0.25868 | 0.02 |
| g__Sebacina | negative | d_Unassigned | -0.25761 | 0.03 |
| g__Sebacina | negative | g__Parabacteroides | -0.25747 | 0.01 |
| g__Colletotrichum | negative | g__Bifidobacterium | -0.25679 | 0.02 |
| c__Sordariomycetes | negative | g__Blautia | -0.25625 | 0.01 |
| d__Bacteria | negative | g__Akkermansia | -0.25317 | 0.03 |
| g__unidentified_1(Sebacinales) | negative | d__Archaea | -0.25277 | 0.02 |
| g__Rhizopogon | negative | d_Unassigned | -0.24979 | 0.03 |
| g__Sebacina | negative | d__Archaea | -0.24906 | 0.03 |
| g__Sebacina | negative | d__Bacteria | -0.24676 | 0.04 |
| g__Russula | negative | p__Basidiomycota | -0.24378 | 0.04 |
| g__Hygrocybe | negative | g__Rhizopogon | -0.24377 | 0.03 |
| g__Aspergillus | negative | p__Ascomycota | -0.24334 | 0.04 |
| g__Wallemia | negative | g__Clostridium_sensu_stricto_1 | -0.24265 | 0.05 |
| g__Cutaneotrichosporon | negative | d__Bacteria | -0.24028 | 0.05 |
| g__unidentified_1(Sebacinales) | negative | g__Parabacteroides | -0.23966 | 0.02 |
| g__Sebacina | negative | g__Faecalibacterium | -0.2386 | 0.03 |
| g__Ruhlandiella | negative | g__Romboutsia | -0.23805 | 0.03 |
| g__Fusicolla | negative | g__Bacteroides | -0.23596 | 0.02 |
| g__Mortierella | negative | g__Paecilomyces | -0.23327 | 0 |
| g__Mortierella | negative | g__Russula | -0.23325 | 0.01 |
| g__Colletotrichum | negative | g__Prevotella | -0.23025 | 0.03 |
| g__Cladosporium | negative | g__Romboutsia | -0.22887 | 0.02 |
| g__Cladosporium | negative | g__Parabacteroides | -0.22639 | 0 |
| g__Ruhlandiella | negative | g__Bifidobacterium | -0.22636 | 0.05 |
| g__Russula | negative | g__Bifidobacterium | -0.22631 | 0.05 |
| g__Alternaria | negative | g__Archaeorhizomyces | -0.22418 | 0.04 |
| g__Bifidobacterium | negative | g__Rothia | -0.22247 | 0.04 |
| g__Fusarium | negative | g__Clostridium_sensu_stricto_1 | -0.22214 | 0.05 |
| g__Epicoccum | negative | g__Blautia | -0.22061 | 0.05 |
| g__Mortierella | negative | g__unidentified_1(Sebacinales) | -0.22024 | 0.04 |
| g__unidentified_1(Sebacinales) | negative | g__Blautia | -0.21985 | 0.04 |
| g__Epicoccum | negative | g__Prevotella | -0.21981 | 0.04 |
| g__Colletotrichum | negative | g__Paecilomyces | -0.2193 | 0.04 |
| g__Cladosporium | negative | g__Cutibacterium | -0.21556 | 0.04 |
| g__Russula | negative | g__Bacteroides | -0.21507 | 0.02 |
| c__Sordariomycetes | negative | g__Bifidobacterium | -0.21376 | 0.04 |
| g__Wallemia | negative | g__Bifidobacterium | -0.21247 | 0.02 |
| g__Epicoccum | negative | g__Rhizopogon | -0.21065 | 0.05 |
| g__Ruhlandiella | negative | f__Lachnospiraceae | -0.20926 | 0.05 |
| g__Parabacteroides | negative | g__Rothia | -0.20435 | 0.04 |
| g__Wallemia | negative | d__Bacteria | -0.20355 | 0.03 |
| g__Sebacina | negative | g__Cutibacterium | -0.20218 | 0.04 |
| g__Sebacina | negative | g__Bacteroides | -0.20088 | 0.05 |
| g__unidentified_1(Sebacinales) | negative | p__Basidiomycota | -0.19758 | 0.04 |
| g__Epicoccum | negative | g__Paecilomyces | -0.19556 | 0.05 |
| g__Cutibacterium | positive | g__Fusobacterium | 0.187758 | 0.05 |
| f__Lachnospiraceae | positive | g__Akkermansia | 0.190812 | 0.04 |
| g__Aspergillus | positive | g__Wallemia | 0.194535 | 0.05 |
| g__Akkermansia | positive | g__Clostridium_sensu_stricto_1 | 0.200721 | 0.04 |
| g__Muribaculum | positive | g__Romboutsia | 0.203295 | 0.03 |
| g__Blautia | positive | g__Clostridium_sensu_stricto_1 | 0.209214 | 0.04 |
| g__Aspergillus | positive | g__Sebacina | 0.218199 | 0.03 |
| d__Bacteria | positive | g__Blautia | 0.219421 | 0.01 |
| g__Epicoccum | positive | p__Basidiomycota | 0.219754 | 0.03 |
| g__Hygrocybe | positive | g__unidentified_21 | 0.222001 | 0.05 |
| f__Lachnospiraceae | positive | g__Romboutsia | 0.222473 | 0.04 |
| g__Aspergillus | positive | g__Cutaneotrichosporon | 0.223308 | 0.04 |
| g__Hygrocybe | positive | g__Mortierella | 0.224689 | 0.02 |
| g__Paecilomyces | positive | g__Rhizopogon | 0.22675 | 0 |
| g__Colletotrichum | positive | g__Ruhlandiella | 0.226874 | 0.04 |
| g__Sebacina | positive | g__unidentified_1(Sebacinales) | 0.233118 | 0.05 |
| g__Blautia | positive | g__Muribaculum | 0.237967 | 0.03 |
| g__Fusicolla | positive | g__unidentified_1(Sebacinales) | 0.23846 | 0.02 |
| g__Fusarium | positive | g__Mortierella | 0.23876 | 0.05 |
| d_Unassigned | positive | g__Bifidobacterium | 0.241766 | 0.03 |
| d_Unassigned | positive | g__Bacteroides | 0.248528 | 0.05 |
| g__Fusobacterium | positive | g__Rothia | 0.250592 | 0.01 |
| g__Blautia | positive | g__Cutibacterium | 0.251054 | 0.03 |
| g__Cutaneotrichosporon | positive | g__Russula | 0.252547 | 0.02 |
| c__Sordariomycetes | positive | g__Sebacina | 0.252602 | 0.01 |
| g__Alternaria | positive | g__Colletotrichum | 0.252772 | 0.01 |
| g__Alternaria | positive | g__unidentified_1(Sebacinales) | 0.253052 | 0.02 |
| g__Alternaria | positive | g__Fusobacterium | 0.254341 | 0.01 |
| g__Bacteroides | positive | g__Cutibacterium | 0.254472 | 0.01 |
| g__Cladosporium | positive | g__Colletotrichum | 0.256431 | 0.02 |
| d__Bacteria | positive | g__Faecalibacterium | 0.259421 | 0.02 |
| g__Fusarium | positive | g__Wallemia | 0.261444 | 0.02 |
| g__Paecilomyces | positive | g__unidentified_21 | 0.263031 | 0.03 |
| d_Unassigned | positive | g__Blautia | 0.263489 | 0.03 |
| p__Ascomycota | positive | d_Unassigned | 0.269434 | 0.04 |
| p__Ascomycota | positive | g__Faecalibacterium | 0.271659 | 0.03 |
| d__Archaea | positive | g__Faecalibacterium | 0.274488 | 0.02 |
| g__Cladosporium | positive | g__Fusarium | 0.274791 | 0 |
| d__Bacteria | positive | g__Romboutsia | 0.275307 | 0 |
| g__Mortierella | positive | g__Rothia | 0.275326 | 0.01 |
| g__Cutibacterium | positive | g__Faecalibacterium | 0.277109 | 0.02 |
| p__Ascomycota | positive | p__Basidiomycota | 0.278094 | 0 |
| g__Clostridium_sensu_stricto_1 | positive | g__Parabacteroides | 0.279608 | 0.01 |
| d_Unassigned | positive | g__Prevotella | 0.280645 | 0.01 |
| g__unidentified_21 | positive | d_Unassigned | 0.285334 | 0 |
| p__Ascomycota | positive | g__Romboutsia | 0.286875 | 0 |
| g__Rhizopogon | positive | g__Ruhlandiella | 0.287159 | 0 |
| g__Rhizopogon | positive | g__Wallemia | 0.289026 | 0.03 |
| g__Paecilomyces | positive | g__Sebacina | 0.290754 | 0.01 |
| g__Mortierella | positive | g__Fusobacterium | 0.293088 | 0 |
| g__Alternaria | positive | g__Mortierella | 0.295448 | 0 |
| p__Ascomycota | positive | d__Bacteria | 0.295582 | 0.01 |
| g__Sebacina | positive | g__Wallemia | 0.29559 | 0.01 |
| g__Hygrocybe | positive | d__Archaea | 0.297561 | 0 |
| d__Bacteria | positive | g__Bacteroides | 0.298156 | 0.01 |
| g__Rhizopogon | positive | g__Russula | 0.299904 | 0 |
| g__Colletotrichum | positive | g__Fusarium | 0.300861 | 0.01 |
| g__Cutibacterium | positive | g__Rothia | 0.301259 | 0 |
| g__Rhizopogon | positive | g__Sebacina | 0.302676 | 0.01 |
| p__Ascomycota | positive | g__Prevotella | 0.303544 | 0 |
| f__Lachnospiraceae | positive | g__Blautia | 0.305778 | 0 |
| g__Epicoccum | positive | g__Fusarium | 0.306275 | 0.01 |
| g__Cutibacterium | positive | g__Prevotella | 0.307095 | 0.02 |
| p__Ascomycota | positive | d__Archaea | 0.30851 | 0.02 |
| g__Parabacteroides | positive | g__Romboutsia | 0.309695 | 0 |
| f__Lachnospiraceae | positive | g__Muribaculum | 0.310543 | 0 |
| d__Archaea | positive | g__Prevotella | 0.312897 | 0.02 |
| g__Prevotella | positive | g__Rothia | 0.315161 | 0.02 |
| g__Bacteroides | positive | g__Muribaculum | 0.316799 | 0.01 |
| c__Sordariomycetes | positive | g__unidentified_1(Sebacinales) | 0.316852 | 0 |
| g__Bacteroides | positive | g__Clostridium_sensu_stricto_1 | 0.321152 | 0 |
| g__Faecalibacterium | positive | g__Parabacteroides | 0.322429 | 0 |
| g__Alternaria | positive | g__Fusicolla | 0.323142 | 0 |
| g__Aspergillus | positive | g__Russula | 0.323629 | 0 |
| g__Ruhlandiella | positive | g__Russula | 0.326174 | 0.01 |
| g__Bifidobacterium | positive | g__Prevotella | 0.328898 | 0 |
| g__Bifidobacterium | positive | g__Blautia | 0.350109 | 0 |
| g__Faecalibacterium | positive | g__Prevotella | 0.356222 | 0 |
| d_Unassigned | positive | g__Romboutsia | 0.357678 | 0 |
| d__Archaea | positive | d__Bacteria | 0.360278 | 0 |
| g__Aspergillus | positive | g__Ruhlandiella | 0.362449 | 0 |
| g__unidentified_1(Sebacinales) | positive | g__Wallemia | 0.364842 | 0 |
| g__Archaeorhizomyces | positive | g__Rhizopogon | 0.36635 | 0 |
| g__Epicoccum | positive | g__unidentified_1(Sebacinales) | 0.366705 | 0 |
| d_Unassigned | positive | g__Faecalibacterium | 0.371311 | 0 |
| g__Ruhlandiella | positive | g__Sebacina | 0.375967 | 0 |
| g__Clostridium_sensu_stricto_1 | positive | g__Cutibacterium | 0.378926 | 0 |
| g__Alternaria | positive | g__Epicoccum | 0.381018 | 0 |
| c__Sordariomycetes | positive | g__Russula | 0.382695 | 0 |
| g__Cladosporium | positive | g__unidentified_1(Sebacinales) | 0.394283 | 0 |
| g__Mortierella | positive | d__Archaea | 0.400463 | 0 |
| g__Cutibacterium | positive | g__Muribaculum | 0.402212 | 0 |
| g__Blautia | positive | g__Faecalibacterium | 0.402884 | 0 |
| g__Colletotrichum | positive | g__unidentified_1(Sebacinales) | 0.404815 | 0 |
| g__Hygrocybe | positive | p__Ascomycota | 0.40656 | 0 |
| g__Muribaculum | positive | g__Parabacteroides | 0.408696 | 0 |
| g__Alternaria | positive | g__Cladosporium | 0.410188 | 0 |
| g__Bifidobacterium | positive | g__Clostridium_sensu_stricto_1 | 0.415596 | 0 |
| g__Archaeorhizomyces | positive | g__Paecilomyces | 0.416913 | 0 |
| f__Lachnospiraceae | positive | g__Bacteroides | 0.421582 | 0 |
| g__Clostridium_sensu_stricto_1 | positive | g__Muribaculum | 0.431058 | 0 |
| d__Archaea | positive | d_Unassigned | 0.432881 | 0 |
| g__Archaeorhizomyces | positive | g__Sebacina | 0.4454 | 0 |
| g__Blautia | positive | g__Romboutsia | 0.455345 | 0 |
| g__Cladosporium | positive | g__Epicoccum | 0.456965 | 0 |
| c__Sordariomycetes | positive | g__Aspergillus | 0.463475 | 0 |
| d__Bacteria | positive | d_Unassigned | 0.468089 | 0 |
| g__Blautia | positive | g__Parabacteroides | 0.470031 | 0 |
| g__Cladosporium | positive | g__Fusicolla | 0.471628 | 0 |
| g__Colletotrichum | positive | g__Epicoccum | 0.472127 | 0 |
| g__Ruhlandiella | positive | g__unidentified_1(Sebacinales) | 0.492208 | 0 |
| g__Ruhlandiella | positive | g__Wallemia | 0.502814 | 0 |
| g__Akkermansia | positive | g__Muribaculum | 0.530268 | 0 |
| g__Faecalibacterium | positive | g__Romboutsia | 0.538759 | 0 |
| g__Bifidobacterium | positive | g__Romboutsia | 0.547287 | 0 |
| k__Fungi | positive | p__Ascomycota | 0.556868 | 0.01 |
| g__Bacteroides | positive | g__Parabacteroides | 0.55758 | 0 |
| g__Bacteroides | positive | g__Bifidobacterium | 0.558282 | 0 |
| g__Bifidobacterium | positive | g__Faecalibacterium | 0.566204 | 0 |
| g__Hygrocybe | positive | k__Fungi | 0.575063 | 0.01 |
| g__Bacteroides | positive | g__Blautia | 0.581286 | 0 |
| k__Fungi | positive | d__Archaea | 0.588844 | 0.02 |
| f__Lachnospiraceae | positive | g__Parabacteroides | 0.593995 | 0 |
| g__Bacteroides | positive | g__Romboutsia | 0.599277 | 0 |
| g__Russula | positive | g__Sebacina | 0.601088 | 0 |
| g__Bacteroides | positive | g__Faecalibacterium | 0.643456 | 0 |

**Table S3 SparCC correlation of bacterial–bacterial network in bile**

| **Source** | **Interaction** | **Target** | **Correlation** | ***P* value** |
| --- | --- | --- | --- | --- |
| d__Bacteria | positive | d_Unassigned | 0.651127 | 0 |
| d__Bacteria | positive | g__Bacteroides | 0.393076 | 0 |
| g__Bacteroides | positive | g__Blautia | 0.503124 | 0 |
| d__Archaea | negative | g__Cutibacterium | -0.3333 | 0.02 |
| d__Archaea | positive | g__Deep_Sea_Euryarchaeotic_Group(DSEG) | 0.351989 | 0.01 |
| g__Bacteroides | negative | g__Deep_Sea_Euryarchaeotic_Group(DSEG) | -0.4391 | 0.02 |
| d__Archaea | positive | g__Faecalibacterium | 0.331096 | 0.02 |
| g__Akkermansia | negative | g__Faecalibacterium | -0.38491 | 0.01 |
| g__Bacteroides | positive | g__Faecalibacterium | 0.316916 | 0.01 |
| g__Bifidobacterium | positive | g__Faecalibacterium | 0.453153 | 0.01 |
| g__Cutibacterium | negative | g__Faecalibacterium | -0.3192 | 0.04 |
| d__Bacteria | negative | g__Muribaculum | -0.39609 | 0 |
| g__Akkermansia | positive | g__Muribaculum | 0.47188 | 0 |
| g__Bifidobacterium | negative | g__Muribaculum | -0.40584 | 0 |
| g__Cutibacterium | positive | g__Muribaculum | 0.465902 | 0 |
| g__Faecalibacterium | negative | g__Muribaculum | -0.41111 | 0.01 |
| g__Bacteroides | positive | g__Parabacteroides | 0.321618 | 0.01 |
| g__Bifidobacterium | negative | g__Parabacteroides | -0.29543 | 0.05 |
| g__Blautia | positive | g__Parabacteroides | 0.323759 | 0.01 |
| g__Bifidobacterium | positive | g__Prevotella | 0.461353 | 0.05 |
| g__Blautia | negative | g__Prevotella | -0.40091 | 0.03 |
| g__Bifidobacterium | positive | g__Romboutsia | 0.335693 | 0.03 |
| g__Faecalibacterium | positive | g__Romboutsia | 0.311131 | 0.03 |
| g__Muribaculum | negative | g__Romboutsia | -0.31241 | 0.05 |
| d__Archaea | negative | g__Rothia | -0.2773 | 0.05 |
| g__Bacteroides | negative | g__Rothia | -0.3181 | 0.04 |
| g__Cutibacterium | positive | g__Rothia | 0.476453 | 0 |
| g__Muribaculum | positive | g__Rothia | 0.404686 | 0 |

**Table S4 SparCC correlation of bacterial–bacterial network in gallstone**

| **Source** | **Interaction** | **Target** | **Correlation** | ***P* value** |
| --- | --- | --- | --- | --- |
| d__Archaea | positive | d__Bacteria | 0.359877 | 0.01 |
| d__Archaea | positive | d_Unassigned | 0.364032 | 0.02 |
| d__Bacteria | negative | f__Lachnospiraceae | -0.2911 | 0.01 |
| d__Archaea | negative | g__Eubacterium_coprostanoligenes_group | -0.27905 | 0.05 |
| d__Archaea | negative | g__Bacteroides | -0.47492 | 0 |
| d_Unassigned | negative | g__Bacteroides | -0.41044 | 0 |
| d__Archaea | negative | g__Blautia | -0.29634 | 0.02 |
| d__Bacteria | positive | g__Clostridium_sensu_stricto_1 | 0.270575 | 0.04 |
| g__Bifidobacterium | positive | g__Clostridium_sensu_stricto_1 | 0.311715 | 0.03 |
| f__Lachnospiraceae | negative | g__Cutibacterium | -0.30908 | 0.03 |
| g__Akkermansia | negative | g__Faecalibacterium | -0.43123 | 0 |
| g__Bacteroides | positive | g__Faecalibacterium | 0.365396 | 0.01 |
| d__Archaea | positive | g__Fusobacterium | 0.393605 | 0 |
| g__Bacteroides | negative | g__Fusobacterium | -0.47089 | 0 |
| g__Cutibacterium | positive | g__Fusobacterium | 0.345916 | 0.03 |
| d__Archaea | negative | g__Muribaculum | -0.34157 | 0.04 |
| d_Unassigned | negative | g__Muribaculum | -0.38171 | 0.02 |
| f__Lachnospiraceae | positive | g__Muribaculum | 0.296394 | 0 |
| g__Akkermansia | positive | g__Muribaculum | 0.368048 | 0.02 |
| g__Bacteroides | positive | g__Muribaculum | 0.451091 | 0 |
| g__Fusobacterium | negative | g__Muribaculum | -0.46602 | 0 |
| f__Lachnospiraceae | positive | g__Parabacteroides | 0.308332 | 0.02 |
| g__Bacteroides | positive | g__Parabacteroides | 0.489288 | 0 |
| g__Muribaculum | positive | g__Parabacteroides | 0.325466 | 0.02 |
| d_Unassigned | positive | g__Prevotella | 0.433813 | 0.04 |
| g__Bacteroides | negative | g__Prevotella | -0.50466 | 0.01 |
| g__Clostridium_sensu_stricto_1 | negative | g__Prevotella | -0.34561 | 0.05 |
| g__Fusobacterium | positive | g__Prevotella | 0.450745 | 0 |
| g__Bacteroides | positive | g__Romboutsia | 0.347815 | 0.05 |
| d__Archaea | positive | g__Rothia | 0.44968 | 0 |
| d_Unassigned | positive | g__Rothia | 0.400283 | 0.01 |
| g__Bacteroides | negative | g__Rothia | -0.52187 | 0 |
| g__Fusobacterium | positive | g__Rothia | 0.459171 | 0.01 |
| g__Muribaculum | negative | g__Rothia | -0.31408 | 0.01 |
| g__Parabacteroides | negative | g__Rothia | -0.40475 | 0.01 |
| g__Prevotella | positive | g__Rothia | 0.613437 | 0.01 |
| g__Muribaculum | positive | g__Ruminococcus | 0.455384 | 0 |

**Table S5 SparCC correlation of fungal–fungal network in bile**

| **Source** | **Interaction** | **Target** | **Correlation** | ***P* value** |
| --- | --- | --- | --- | --- |
| c__Sordariomycetes | negative | g__Archaeorhizomyces | -0.43122 | 0 |
| g__Alternaria | negative | g__Archaeorhizomyces | -0.31441 | 0.05 |
| c__Sordariomycetes | positive | g__Aspergillus | 0.427305 | 0.02 |
| g__Archaeorhizomyces | negative | g__Aspergillus | -0.36745 | 0 |
| g__Alternaria | positive | g__Cladosporium | 0.4002 | 0.01 |
| g__Archaeorhizomyces | positive | g__Conocybe | 0.591145 | 0 |
| g__Alternaria | positive | g__Epicoccum | 0.519967 | 0 |
| g__Cladosporium | positive | g__Epicoccum | 0.367328 | 0.01 |
| g__Colletotrichum | positive | g__Epicoccum | 0.403363 | 0.01 |
| g__Conocybe | negative | g__Epicoccum | -0.3504 | 0.02 |
| g__Colletotrichum | positive | g__Fusarium | 0.32866 | 0.02 |
| g__Colletotrichum | negative | g__Paecilomyces | -0.35284 | 0.02 |
| g__Conocybe | positive | g__Paecilomyces | 0.394876 | 0.01 |
| g__Fusarium | negative | g__Paecilomyces | -0.41169 | 0.02 |
| g__Archaeorhizomyces | positive | g__Rhizopogon | 0.464768 | 0.01 |
| g__Epicoccum | negative | g__Rhizopogon | -0.48171 | 0.01 |
| g__Aspergillus | positive | g__Ruhlandiella | 0.2952 | 0.04 |
| g__Mortierella | negative | g__Russula | -0.36975 | 0.03 |
| g__Archaeorhizomyces | positive | g__Sebacina | 0.527337 | 0 |
| g__Paecilomyces | positive | g__Sebacina | 0.354214 | 0.05 |
| g__Russula | positive | g__Sebacina | 0.561295 | 0 |
| g__Paecilomyces | positive | g__unidentified_21 | 0.434472 | 0.01 |
| g__Hygrocybe | positive | k__Fungi | 0.539021 | 0.02 |
| g__Hygrocybe | positive | p__Ascomycota | 0.559151 | 0 |
| g__Ruhlandiella | negative | p__Ascomycota | -0.38833 | 0.05 |
| g__Sebacina | negative | p__Ascomycota | -0.35391 | 0.05 |
| k__Fungi | positive | p__Ascomycota | 0.847657 | 0 |
| p__Ascomycota | positive | p__Basidiomycota | 0.368978 | 0.04 |

**Table S6 SparCC correlation of fungal–fungal network in gallstone**

| **Source** | **Interaction** | **Target** | **Correlation** | ***P* value** |
| --- | --- | --- | --- | --- |
| c__Sordariomycetes | negative | f__Didymellaceae | -0.48135 | 0 |
| c__Sordariomycetes | positive | g__Aspergillus | 0.427045 | 0.01 |
| f__Didymellaceae | positive | g__Cladosporium | 0.420342 | 0.01 |
| g__Aspergillus | negative | g__Epicoccum | -0.41728 | 0.02 |
| g__Cladosporium | positive | g__Epicoccum | 0.393903 | 0 |
| g__Colletotrichum | positive | g__Epicoccum | 0.419614 | 0.01 |
| g__Alternaria | positive | g__Fusarium | 0.553225 | 0 |
| g__Aspergillus | negative | g__Fusarium | -0.38195 | 0.01 |
| g__Cladosporium | positive | g__Fusicolla | 0.562869 | 0 |
| g__Archaeorhizomyces | positive | g__Hygrocybe | 0.351225 | 0.04 |
| g__Fusarium | positive | g__Hygrocybe | 0.474215 | 0 |
| g__Fusicolla | negative | g__Hygrocybe | -0.31739 | 0.03 |
| g__Archaeorhizomyces | negative | g__Inocybe | -0.31804 | 0.04 |
| g__Fusarium | negative | g__Inocybe | -0.35434 | 0.02 |
| g__Archaeorhizomyces | negative | g__Lactarius | -0.39048 | 0.01 |
| g__Fusicolla | positive | g__Lactarius | 0.332054 | 0.04 |
| g__Hygrocybe | negative | g__Lactarius | -0.35683 | 0 |
| g__Alternaria | positive | g__Mortierella | 0.448307 | 0.01 |
| g__Aspergillus | negative | g__Mortierella | -0.45701 | 0 |
| g__Fusarium | positive | g__Mortierella | 0.566504 | 0 |
| g__Hygrocybe | positive | g__Mortierella | 0.53909 | 0 |
| g__Archaeorhizomyces | positive | g__Paecilomyces | 0.593446 | 0 |
| g__Aspergillus | positive | g__Paecilomyces | 0.30878 | 0.05 |
| g__Colletotrichum | negative | g__Polypaecilum | -0.4407 | 0.02 |
| g__Fusicolla | positive | g__Polypaecilum | 0.322561 | 0.04 |
| g__Inocybe | positive | g__Polypaecilum | 0.424177 | 0.02 |
| g__Paecilomyces | negative | g__Polypaecilum | -0.34975 | 0.01 |
| g__Inocybe | positive | g__Pseudombrophila | 0.372593 | 0.03 |
| g__Alternaria | negative | g__Rhizopogon | -0.49492 | 0 |
| g__Fusarium | negative | g__Rhizopogon | -0.44244 | 0 |
| g__Hygrocybe | negative | g__Rhizopogon | -0.31724 | 0.05 |
| g__Fusarium | negative | g__Ruhlandiella | -0.43556 | 0 |
| g__Hygrocybe | negative | g__Ruhlandiella | -0.45848 | 0 |
| g__Mortierella | negative | g__Ruhlandiella | -0.46033 | 0 |
| g__Aspergillus | positive | g__Russula | 0.388288 | 0.04 |
| g__Cladosporium | negative | g__Russula | -0.44434 | 0.02 |
| g__Epicoccum | negative | g__Russula | -0.32582 | 0.05 |
| g__Fusicolla | negative | g__Russula | -0.33876 | 0.02 |
| g__Inocybe | positive | g__Russula | 0.471633 | 0 |
| g__Alternaria | negative | g__Sebacina | -0.52908 | 0 |
| g__Colletotrichum | negative | g__Sebacina | -0.34296 | 0.04 |
| g__Russula | positive | g__Sebacina | 0.426044 | 0 |
| f__Didymellaceae | positive | g__Tausonia | 0.3463 | 0.05 |
| g__Fusarium | negative | g__Tausonia | -0.42552 | 0 |
| f__Didymellaceae | positive | g__unidentified_01 | 0.349701 | 0.05 |
| g__Cladosporium | positive | g__unidentified_01 | 0.459792 | 0 |
| g__Inocybe | positive | g__unidentified_01 | 0.627126 | 0 |
| g__Mortierella | negative | g__unidentified_01 | -0.42503 | 0.02 |
| g__Ruhlandiella | positive | g__unidentified_01 | 0.399884 | 0 |
| g__Sebacina | negative | g__unidentified_02 | -0.2839 | 0.04 |
| g__unidentified_01 | positive | g__unidentified_02 | 0.468133 | 0 |
| g__Hygrocybe | negative | g__unidentified_05 | -0.40898 | 0 |
| g__Mortierella | negative | g__unidentified_05 | -0.35965 | 0.02 |
| g__Ruhlandiella | positive | g__unidentified_05 | 0.422416 | 0.01 |
| g__Russula | negative | g__unidentified_05 | -0.34021 | 0.01 |
| g__unidentified_01 | positive | g__unidentified_05 | 0.39627 | 0 |
| g__unidentified_02 | positive | g__unidentified_05 | 0.318917 | 0.04 |
| g__Cladosporium | negative | g__unidentified_06 | -0.46838 | 0.01 |
| g__Russula | positive | g__unidentified_06 | 0.406385 | 0 |
| g__Cutaneotrichosporon | negative | g__Wallemia | -0.32396 | 0.04 |
| g__Ruhlandiella | positive | g__Wallemia | 0.450679 | 0 |
| g__unidentified_02 | positive | g__Wallemia | 0.395947 | 0 |
| g__unidentified_06 | positive | g__Wallemia | 0.43167 | 0.01 |
| g__Cutaneotrichosporon | negative | g__Wilcoxina | -0.36824 | 0 |
| g__Epicoccum | negative | g__Wilcoxina | -0.32276 | 0.04 |
| g__Wallemia | positive | g__Wilcoxina | 0.359078 | 0.03 |
| g__Archaeorhizomyces | positive | k__Fungi | 0.423751 | 0.05 |
| g__Hygrocybe | positive | k__Fungi | 0.693517 | 0 |
| g__Inocybe | negative | k__Fungi | -0.48484 | 0.02 |
| g__Lactarius | negative | k__Fungi | -0.64291 | 0.01 |
| g__Mortierella | positive | k__Fungi | 0.58455 | 0.05 |
| g__Ruhlandiella | negative | k__Fungi | -0.56266 | 0.03 |
| g__Cladosporium | negative | o__Mortierellales | -0.33501 | 0.02 |
| g__Colletotrichum | negative | o__Mortierellales | -0.37729 | 0 |
| g__Epicoccum | negative | o__Mortierellales | -0.32634 | 0.05 |
| g__unidentified_06 | positive | o__Mortierellales | 0.333097 | 0.04 |
| g__Archaeorhizomyces | positive | p__Ascomycota | 0.334999 | 0.05 |
| g__Hygrocybe | positive | p__Ascomycota | 0.552043 | 0 |
| g__Inocybe | negative | p__Ascomycota | -0.3872 | 0.02 |
| g__Lactarius | negative | p__Ascomycota | -0.39629 | 0.03 |
| g__Mortierella | positive | p__Ascomycota | 0.473807 | 0.01 |
| g__Ruhlandiella | negative | p__Ascomycota | -0.40584 | 0.02 |
| g__unidentified_01 | negative | p__Ascomycota | -0.49656 | 0.01 |
| g__unidentified_02 | negative | p__Ascomycota | -0.42128 | 0.02 |
| g__unidentified_05 | negative | p__Ascomycota | -0.37221 | 0.04 |
| k__Fungi | positive | p__Ascomycota | 0.765781 | 0.01 |
| f__Didymellaceae | negative | p__Basidiomycota | -0.32062 | 0.01 |
| g__Archaeorhizomyces | positive | p__Basidiomycota | 0.326898 | 0.04 |
| g__Inocybe | negative | p__Basidiomycota | -0.63807 | 0 |
| g__Mortierella | positive | p__Basidiomycota | 0.319633 | 0.04 |
| g__Pseudombrophila | negative | p__Basidiomycota | -0.40058 | 0.01 |
| g__unidentified_01 | negative | p__Basidiomycota | -0.51844 | 0 |
| k__Fungi | positive | p__Basidiomycota | 0.542522 | 0.02 |
| p__Ascomycota | positive | p__Basidiomycota | 0.590987 | 0 |

**Table S7 SparCC correlation of bacterial–fungal network in bile**

| **Source** | **Interaction** | **Target** | **Correlation** | ***P* value** |
| --- | --- | --- | --- | --- |
| d__Archaea | positive | d_Unassigned | 0.403393 | 0.03 |
| d__Bacteria | positive | d_Unassigned | 0.619934 | 0 |
| c__Sordariomycetes | positive | g__Alternaria | 0.305958 | 0.04 |
| d__Archaea | negative | g__Archaeorhizomyces | -0.41488 | 0.02 |
| d__Bacteria | negative | g__Archaeorhizomyces | -0.32058 | 0.03 |
| d_Unassigned | negative | g__Archaeorhizomyces | -0.52271 | 0 |
| c__Sordariomycetes | positive | g__Aspergillus | 0.430696 | 0.01 |
| c__Sordariomycetes | negative | g__Bacteroides | -0.29159 | 0.05 |
| d__Bacteria | positive | g__Bacteroides | 0.511123 | 0 |
| d_Unassigned | positive | g__Bacteroides | 0.499926 | 0 |
| g__Bacteroides | positive | g__Bifidobacterium | 0.438297 | 0 |
| d_Unassigned | positive | g__Blautia | 0.375861 | 0.02 |
| g__Bacteroides | positive | g__Blautia | 0.646139 | 0 |
| g__Alternaria | positive | g__Cladosporium | 0.447538 | 0.01 |
| g__Bifidobacterium | positive | g__Clostridium_sensu_stricto_1 | 0.319802 | 0.03 |
| d_Unassigned | negative | g__Conocybe | -0.36419 | 0.03 |
| g__Archaeorhizomyces | positive | g__Conocybe | 0.616076 | 0 |
| d__Archaea | positive | g__Deep_Sea_Euryarchaeotic_Group(DSEG) | 0.399833 | 0.02 |
| g__Alternaria | positive | g__Epicoccum | 0.550166 | 0 |
| g__Cladosporium | positive | g__Epicoccum | 0.353264 | 0.01 |
| g__Colletotrichum | positive | g__Epicoccum | 0.463786 | 0 |
| d__Archaea | positive | g__Faecalibacterium | 0.496405 | 0 |
| d__Bacteria | positive | g__Faecalibacterium | 0.408762 | 0 |
| d_Unassigned | positive | g__Faecalibacterium | 0.480379 | 0.02 |
| g__Bacteroides | positive | g__Faecalibacterium | 0.614363 | 0 |
| g__Bifidobacterium | positive | g__Faecalibacterium | 0.578238 | 0 |
| g__Blautia | positive | g__Faecalibacterium | 0.324753 | 0.04 |
| g__Colletotrichum | negative | g__Faecalibacterium | -0.304 | 0.04 |
| g__Epicoccum | negative | g__Faecalibacterium | -0.41055 | 0 |
| d__Bacteria | positive | g__Fusarium | 0.348499 | 0.01 |
| g__Conocybe | negative | g__Fusarium | -0.3133 | 0.04 |
| c__Sordariomycetes | negative | g__Muribaculum | -0.30821 | 0.05 |
| g__Akkermansia | positive | g__Muribaculum | 0.583187 | 0 |
| g__Clostridium_sensu_stricto_1 | positive | g__Muribaculum | 0.383294 | 0 |
| g__Cutibacterium | positive | g__Muribaculum | 0.589003 | 0 |
| g__Conocybe | positive | g__Paecilomyces | 0.385567 | 0.01 |
| g__Fusarium | negative | g__Paecilomyces | -0.424 | 0.01 |
| d__Archaea | positive | g__Prevotella | 0.327754 | 0.04 |
| d_Unassigned | positive | g__Prevotella | 0.40662 | 0.03 |
| g__Bacteroides | positive | g__Prevotella | 0.332025 | 0.02 |
| g__Bifidobacterium | positive | g__Prevotella | 0.59476 | 0 |
| g__Clostridium_sensu_stricto_1 | positive | g__Prevotella | 0.352581 | 0.04 |
| g__Colletotrichum | negative | g__Prevotella | -0.28068 | 0.05 |
| g__Cutibacterium | positive | g__Prevotella | 0.389916 | 0.02 |
| g__Faecalibacterium | positive | g__Prevotella | 0.526257 | 0 |
| d_Unassigned | negative | g__Rhizopogon | -0.45196 | 0.01 |
| g__Archaeorhizomyces | positive | g__Rhizopogon | 0.526268 | 0 |
| g__Conocybe | positive | g__Rhizopogon | 0.338621 | 0.01 |
| g__Cutibacterium | negative | g__Rhizopogon | -0.29094 | 0.03 |
| g__Epicoccum | negative | g__Rhizopogon | -0.33353 | 0.05 |
| d__Bacteria | positive | g__Romboutsia | 0.376226 | 0 |
| d_Unassigned | positive | g__Romboutsia | 0.327131 | 0.05 |
| g__Archaeorhizomyces | negative | g__Romboutsia | -0.28975 | 0.05 |
| g__Bacteroides | positive | g__Romboutsia | 0.51308 | 0 |
| g__Bifidobacterium | positive | g__Romboutsia | 0.472299 | 0 |
| g__Blautia | positive | g__Romboutsia | 0.445717 | 0.02 |
| g__Faecalibacterium | positive | g__Romboutsia | 0.505304 | 0.01 |
| g__Prevotella | positive | g__Romboutsia | 0.342175 | 0.04 |
| d__Bacteria | negative | g__Rothia | -0.32275 | 0.05 |
| g__Cutibacterium | positive | g__Rothia | 0.447067 | 0 |
| g__Muribaculum | positive | g__Rothia | 0.441022 | 0.01 |
| g__Prevotella | negative | g__Ruhlandiella | -0.37434 | 0.01 |
| c__Sordariomycetes | positive | g__Russula | 0.455444 | 0.01 |
| d__Archaea | negative | g__Russula | -0.29418 | 0.05 |
| g__Archaeorhizomyces | positive | g__Russula | 0.363537 | 0.03 |
| g__Bifidobacterium | negative | g__Russula | -0.43748 | 0.01 |
| g__Faecalibacterium | negative | g__Russula | -0.30995 | 0.04 |
| g__Prevotella | negative | g__Russula | -0.39959 | 0.01 |
| g__Ruhlandiella | positive | g__Russula | 0.305694 | 0.03 |
| d__Archaea | negative | g__Sebacina | -0.35575 | 0.04 |
| d_Unassigned | negative | g__Sebacina | -0.36359 | 0.01 |
| g__Archaeorhizomyces | positive | g__Sebacina | 0.606105 | 0 |
| g__Bacteroides | negative | g__Sebacina | -0.35578 | 0.04 |
| g__Conocybe | positive | g__Sebacina | 0.385765 | 0.01 |
| g__Faecalibacterium | negative | g__Sebacina | -0.346 | 0.04 |
| g__Paecilomyces | positive | g__Sebacina | 0.408528 | 0 |
| g__Prevotella | negative | g__Sebacina | -0.48633 | 0 |
| g__Rhizopogon | positive | g__Sebacina | 0.326835 | 0.04 |
| g__Ruhlandiella | positive | g__Sebacina | 0.319675 | 0.03 |
| g__Russula | positive | g__Sebacina | 0.65446 | 0 |
| g__Muribaculum | negative | g__unidentified_21 | -0.32773 | 0.03 |
| g__Paecilomyces | positive | g__unidentified_21 | 0.443387 | 0.02 |
| g__Cladosporium | negative | k__Fungi | -0.4829 | 0.04 |
| d__Archaea | positive | p__Ascomycota | 0.414556 | 0.02 |
| g__Bifidobacterium | positive | p__Ascomycota | 0.32339 | 0.05 |
| g__Cladosporium | negative | p__Ascomycota | -0.46716 | 0 |
| g__Epicoccum | negative | p__Ascomycota | -0.3274 | 0.03 |
| g__Faecalibacterium | positive | p__Ascomycota | 0.5035 | 0.01 |
| g__Fusarium | negative | p__Ascomycota | -0.3634 | 0.02 |
| g__Hygrocybe | positive | p__Ascomycota | 0.515865 | 0 |
| g__Prevotella | positive | p__Ascomycota | 0.389128 | 0.04 |
| g__Ruhlandiella | negative | p__Ascomycota | -0.44735 | 0.02 |
| k__Fungi | positive | p__Ascomycota | 0.642233 | 0.02 |
| g__Cladosporium | negative | p__Basidiomycota | -0.31387 | 0.05 |

**Table S8 SparCC correlation of bacterial–fungal network in gallstone**

| **Source** | **Interaction** | **Target** | **Correlation** | ***P* value** |
| --- | --- | --- | --- | --- |
| d__Archaea | positive | d__Bacteria | 0.362127 | 0.03 |
| d__Archaea | positive | d_Unassigned | 0.358096 | 0.02 |
| d_Unassigned | negative | f__Didymellaceae | -0.328 | 0.02 |
| c__Sordariomycetes | negative | f__Lachnospiraceae | -0.42008 | 0.01 |
| f__Didymellaceae | negative | f__Lachnospiraceae | -0.37724 | 0.01 |
| c__Sordariomycetes | negative | g__Eubacterium_coprostanoligenes_group | -0.38975 | 0.02 |
| f__Didymellaceae | negative | g__Eubacterium_coprostanoligenes_group | -0.34886 | 0 |
| f__Lachnospiraceae | positive | g__Eubacterium_coprostanoligenes_group | 0.60426 | 0 |
| c__Sordariomycetes | negative | g__Akkermansia | -0.44177 | 0 |
| f__Lachnospiraceae | positive | g__Akkermansia | 0.440958 | 0 |
| f__Didymellaceae | positive | g__Alternaria | 0.306157 | 0.04 |
| g__Akkermansia | negative | g__Alternaria | -0.39796 | 0.01 |
| c__Sordariomycetes | positive | g__Aspergillus | 0.525412 | 0 |
| c__Sordariomycetes | negative | g__Bacteroides | -0.41374 | 0 |
| f__Lachnospiraceae | positive | g__Bacteroides | 0.605732 | 0 |
| g__Akkermansia | positive | g__Bacteroides | 0.439381 | 0.01 |
| c__Sordariomycetes | negative | g__Bifidobacterium | -0.36391 | 0.03 |
| f__Lachnospiraceae | positive | g__Bifidobacterium | 0.426948 | 0 |
| g__Akkermansia | positive | g__Bifidobacterium | 0.523261 | 0 |
| g__Bacteroides | positive | g__Bifidobacterium | 0.691426 | 0 |
| f__Lachnospiraceae | positive | g__Blautia | 0.485688 | 0 |
| g__Akkermansia | positive | g__Blautia | 0.307156 | 0.03 |
| g__Bacteroides | positive | g__Blautia | 0.611192 | 0 |
| g__Bifidobacterium | positive | g__Blautia | 0.593528 | 0 |
| f__Didymellaceae | positive | g__Cladosporium | 0.510524 | 0 |
| f__Lachnospiraceae | negative | g__Cladosporium | -0.45779 | 0.01 |
| g__Akkermansia | negative | g__Cladosporium | -0.3505 | 0.01 |
| g__Alternaria | positive | g__Cladosporium | 0.352365 | 0.02 |
| g__Bacteroides | negative | g__Cladosporium | -0.48832 | 0 |
| g__Bifidobacterium | negative | g__Cladosporium | -0.43597 | 0 |
| d__Bacteria | positive | g__Clostridium_sensu_stricto_1 | 0.416944 | 0.01 |
| f__Didymellaceae | negative | g__Clostridium_sensu_stricto_1 | -0.50114 | 0.01 |
| f__Lachnospiraceae | positive | g__Clostridium_sensu_stricto_1 | 0.34311 | 0.02 |
| g__Akkermansia | positive | g__Clostridium_sensu_stricto_1 | 0.445567 | 0.01 |
| g__Bacteroides | positive | g__Clostridium_sensu_stricto_1 | 0.629138 | 0 |
| g__Bifidobacterium | positive | g__Clostridium_sensu_stricto_1 | 0.650373 | 0 |
| g__Blautia | positive | g__Clostridium_sensu_stricto_1 | 0.435653 | 0 |
| g__Cladosporium | negative | g__Clostridium_sensu_stricto_1 | -0.35343 | 0.03 |
| d__Bacteria | positive | g__Cutibacterium | 0.370442 | 0 |
| f__Didymellaceae | negative | g__Cutibacterium | -0.46535 | 0 |
| g__Bacteroides | positive | g__Cutibacterium | 0.433784 | 0 |
| g__Bifidobacterium | positive | g__Cutibacterium | 0.417599 | 0 |
| g__Blautia | positive | g__Cutibacterium | 0.448433 | 0.01 |
| g__Clostridium_sensu_stricto_1 | positive | g__Cutibacterium | 0.533904 | 0 |
| g__Bacteroides | negative | g__Epicoccum | -0.3546 | 0.02 |
| g__Bifidobacterium | negative | g__Epicoccum | -0.39588 | 0.04 |
| g__Blautia | negative | g__Epicoccum | -0.38316 | 0 |
| g__Cladosporium | positive | g__Epicoccum | 0.519281 | 0 |
| g__Colletotrichum | positive | g__Epicoccum | 0.484904 | 0 |
| g__Cutibacterium | negative | g__Epicoccum | -0.30032 | 0.04 |
| d_Unassigned | positive | g__Faecalibacterium | 0.293659 | 0.05 |
| f__Lachnospiraceae | positive | g__Faecalibacterium | 0.394701 | 0 |
| g__Bacteroides | positive | g__Faecalibacterium | 0.692956 | 0 |
| g__Bifidobacterium | positive | g__Faecalibacterium | 0.495314 | 0 |
| g__Blautia | positive | g__Faecalibacterium | 0.534123 | 0 |
| g__Cladosporium | negative | g__Faecalibacterium | -0.45204 | 0.01 |
| g__Clostridium_sensu_stricto_1 | positive | g__Faecalibacterium | 0.379953 | 0.01 |
| g__Cutibacterium | positive | g__Faecalibacterium | 0.584326 | 0 |
| g__Epicoccum | negative | g__Faecalibacterium | -0.50817 | 0 |
| d__Archaea | positive | g__Fusarium | 0.344694 | 0.03 |
| f__Lachnospiraceae | negative | g__Fusarium | -0.40198 | 0.03 |
| g__Akkermansia | negative | g__Fusarium | -0.40386 | 0.02 |
| g__Alternaria | positive | g__Fusarium | 0.60901 | 0 |
| g__Bacteroides | negative | g__Fusarium | -0.51325 | 0.01 |
| g__Bifidobacterium | negative | g__Fusarium | -0.47649 | 0 |
| g__Blautia | negative | g__Fusarium | -0.33041 | 0.02 |
| g__Cladosporium | positive | g__Fusarium | 0.399735 | 0.04 |
| g__Colletotrichum | positive | g__Fusarium | 0.286957 | 0.05 |
| g__Epicoccum | positive | g__Fusarium | 0.328819 | 0.03 |
| g__Faecalibacterium | negative | g__Fusarium | -0.3925 | 0 |
| f__Didymellaceae | positive | g__Fusicolla | 0.37311 | 0.02 |
| f__Lachnospiraceae | negative | g__Fusicolla | -0.50085 | 0 |
| g__Akkermansia | negative | g__Fusicolla | -0.3024 | 0.03 |
| g__Alternaria | positive | g__Fusicolla | 0.417746 | 0 |
| g__Bacteroides | negative | g__Fusicolla | -0.36712 | 0.03 |
| g__Cladosporium | positive | g__Fusicolla | 0.688683 | 0 |
| g__Fusarium | positive | g__Fusicolla | 0.440629 | 0.01 |
| d__Archaea | positive | g__Fusobacterium | 0.294427 | 0.03 |
| g__Alternaria | positive | g__Fusobacterium | 0.31916 | 0.05 |
| g__Aspergillus | negative | g__Fusobacterium | -0.44306 | 0 |
| d__Archaea | positive | g__Hygrocybe | 0.439054 | 0 |
| d__Bacteria | positive | g__Hygrocybe | 0.363288 | 0.05 |
| g__Fusarium | positive | g__Hygrocybe | 0.314689 | 0.04 |
| g__Fusicolla | negative | g__Hygrocybe | -0.33142 | 0.02 |
| c__Sordariomycetes | positive | g__Inocybe | 0.303693 | 0.04 |
| f__Didymellaceae | positive | g__Inocybe | 0.413322 | 0.01 |
| f__Lachnospiraceae | negative | g__Inocybe | -0.30279 | 0.05 |
| g__Aspergillus | positive | g__Inocybe | 0.385428 | 0.03 |
| g__Bacteroides | negative | g__Inocybe | -0.43661 | 0.01 |
| g__Blautia | negative | g__Inocybe | -0.38894 | 0 |
| g__Clostridium_sensu_stricto_1 | negative | g__Inocybe | -0.42671 | 0.01 |
| g__Cutibacterium | negative | g__Inocybe | -0.31578 | 0.04 |
| g__Faecalibacterium | negative | g__Inocybe | -0.34177 | 0.04 |
| d__Archaea | negative | g__Lactarius | -0.28262 | 0.04 |
| d__Bacteria | negative | g__Lactarius | -0.387 | 0.03 |
| f__Didymellaceae | positive | g__Lactarius | 0.361181 | 0.03 |
| f__Lachnospiraceae | negative | g__Lactarius | -0.38794 | 0.04 |
| g__Archaeorhizomyces | negative | g__Lactarius | -0.32123 | 0.03 |
| g__Blautia | negative | g__Lactarius | -0.35441 | 0.03 |
| g__Clostridium_sensu_stricto_1 | negative | g__Lactarius | -0.3603 | 0.05 |
| g__Cutibacterium | negative | g__Lactarius | -0.4056 | 0.01 |
| g__Fusicolla | positive | g__Lactarius | 0.450549 | 0 |
| g__Inocybe | positive | g__Lactarius | 0.435162 | 0.03 |
| d__Archaea | positive | g__Mortierella | 0.524842 | 0 |
| g__Alternaria | positive | g__Mortierella | 0.427603 | 0 |
| g__Aspergillus | negative | g__Mortierella | -0.44194 | 0 |
| g__Fusarium | positive | g__Mortierella | 0.508647 | 0 |
| g__Fusobacterium | positive | g__Mortierella | 0.513999 | 0 |
| g__Hygrocybe | positive | g__Mortierella | 0.485638 | 0 |
| f__Lachnospiraceae | positive | g__Muribaculum | 0.567223 | 0 |
| g__Akkermansia | positive | g__Muribaculum | 0.58493 | 0 |
| g__Alternaria | negative | g__Muribaculum | -0.49622 | 0 |
| g__Bacteroides | positive | g__Muribaculum | 0.687909 | 0 |
| g__Bifidobacterium | positive | g__Muribaculum | 0.426547 | 0 |
| g__Blautia | positive | g__Muribaculum | 0.430899 | 0 |
| g__Cladosporium | negative | g__Muribaculum | -0.33746 | 0.05 |
| g__Clostridium_sensu_stricto_1 | positive | g__Muribaculum | 0.517161 | 0 |
| g__Cutibacterium | positive | g__Muribaculum | 0.306401 | 0.04 |
| g__Fusarium | negative | g__Muribaculum | -0.48659 | 0.01 |
| g__Fusobacterium | negative | g__Muribaculum | -0.35024 | 0.02 |
| g__Mortierella | negative | g__Muribaculum | -0.49706 | 0 |
| d__Bacteria | negative | g__Nothophoma | -0.33571 | 0.05 |
| d_Unassigned | negative | g__Nothophoma | -0.37865 | 0.02 |
| g__Clostridium_sensu_stricto_1 | negative | g__Nothophoma | -0.29516 | 0.03 |
| g__Cutibacterium | negative | g__Nothophoma | -0.41876 | 0 |
| d__Archaea | negative | g__Paecilomyces | -0.32121 | 0.04 |
| g__Archaeorhizomyces | positive | g__Paecilomyces | 0.519616 | 0 |
| g__Mortierella | negative | g__Paecilomyces | -0.33836 | 0.03 |
| c__Sordariomycetes | negative | g__Parabacteroides | -0.46264 | 0 |
| f__Lachnospiraceae | positive | g__Parabacteroides | 0.564209 | 0 |
| g__Akkermansia | positive | g__Parabacteroides | 0.398488 | 0 |
| g__Bacteroides | positive | g__Parabacteroides | 0.717526 | 0 |
| g__Bifidobacterium | positive | g__Parabacteroides | 0.438026 | 0 |
| g__Blautia | positive | g__Parabacteroides | 0.525854 | 0.01 |
| g__Cladosporium | negative | g__Parabacteroides | -0.37469 | 0.01 |
| g__Clostridium_sensu_stricto_1 | positive | g__Parabacteroides | 0.388033 | 0.03 |
| g__Faecalibacterium | positive | g__Parabacteroides | 0.533603 | 0 |
| g__Fusarium | negative | g__Parabacteroides | -0.45704 | 0 |
| g__Fusicolla | negative | g__Parabacteroides | -0.35154 | 0.04 |
| g__Inocybe | negative | g__Parabacteroides | -0.41696 | 0.01 |
| g__Muribaculum | positive | g__Parabacteroides | 0.56589 | 0 |
| d_Unassigned | positive | g__Prevotella | 0.386997 | 0.05 |
| g__Cutibacterium | positive | g__Prevotella | 0.380131 | 0.03 |
| g__Fusobacterium | positive | g__Prevotella | 0.381537 | 0.01 |
| g__Lactarius | negative | g__Prevotella | -0.37841 | 0.02 |
| d__Archaea | negative | g__Rhizopogon | -0.42877 | 0.01 |
| d_Unassigned | negative | g__Rhizopogon | -0.28855 | 0.05 |
| g__Fusarium | negative | g__Rhizopogon | -0.28913 | 0.05 |
| g__Fusobacterium | negative | g__Rhizopogon | -0.31862 | 0.04 |
| g__Hygrocybe | negative | g__Rhizopogon | -0.42624 | 0 |
| g__Mortierella | negative | g__Rhizopogon | -0.31589 | 0.03 |
| g__Nothophoma | positive | g__Rhizopogon | 0.346733 | 0.05 |
| f__Lachnospiraceae | positive | g__Romboutsia | 0.521297 | 0 |
| g__Akkermansia | positive | g__Romboutsia | 0.352962 | 0.02 |
| g__Bacteroides | positive | g__Romboutsia | 0.671287 | 0 |
| g__Bifidobacterium | positive | g__Romboutsia | 0.611529 | 0 |
| g__Blautia | positive | g__Romboutsia | 0.5354 | 0 |
| g__Cladosporium | negative | g__Romboutsia | -0.39517 | 0.01 |
| g__Clostridium_sensu_stricto_1 | positive | g__Romboutsia | 0.455211 | 0 |
| g__Colletotrichum | negative | g__Romboutsia | -0.33648 | 0.05 |
| g__Cutibacterium | positive | g__Romboutsia | 0.46154 | 0 |
| g__Epicoccum | negative | g__Romboutsia | -0.52064 | 0 |
| g__Faecalibacterium | positive | g__Romboutsia | 0.583799 | 0 |
| g__Fusarium | negative | g__Romboutsia | -0.45228 | 0 |
| g__Fusicolla | negative | g__Romboutsia | -0.33931 | 0.01 |
| g__Muribaculum | positive | g__Romboutsia | 0.526355 | 0 |
| g__Nothophoma | negative | g__Romboutsia | -0.40511 | 0 |
| g__Parabacteroides | positive | g__Romboutsia | 0.520208 | 0 |
| d__Archaea | positive | g__Rothia | 0.346977 | 0.03 |
| d_Unassigned | positive | g__Rothia | 0.304453 | 0.05 |
| g__Bacteroides | negative | g__Rothia | -0.30045 | 0.04 |
| g__Fusobacterium | positive | g__Rothia | 0.342172 | 0.03 |
| g__Mortierella | positive | g__Rothia | 0.498244 | 0 |
| g__Prevotella | positive | g__Rothia | 0.539268 | 0 |
| d__Bacteria | negative | g__Ruhlandiella | -0.37989 | 0.02 |
| f__Didymellaceae | positive | g__Ruhlandiella | 0.476592 | 0.01 |
| f__Lachnospiraceae | negative | g__Ruhlandiella | -0.39387 | 0.02 |
| g__Aspergillus | positive | g__Ruhlandiella | 0.362352 | 0.02 |
| g__Blautia | negative | g__Ruhlandiella | -0.49821 | 0 |
| g__Cutibacterium | negative | g__Ruhlandiella | -0.47719 | 0 |
| g__Hygrocybe | negative | g__Ruhlandiella | -0.38841 | 0.04 |
| g__Inocybe | positive | g__Ruhlandiella | 0.493257 | 0 |
| g__Lactarius | positive | g__Ruhlandiella | 0.518988 | 0 |
| g__Mortierella | negative | g__Ruhlandiella | -0.33564 | 0.03 |
| g__Nothophoma | positive | g__Ruhlandiella | 0.369666 | 0.01 |
| g__Prevotella | negative | g__Ruhlandiella | -0.42975 | 0 |
| g__Rhizopogon | positive | g__Ruhlandiella | 0.314366 | 0.01 |
| g__Rothia | negative | g__Ruhlandiella | -0.35264 | 0.05 |
| c__Sordariomycetes | negative | g__Ruminococcus | -0.34106 | 0.01 |
| f__Lachnospiraceae | positive | g__Ruminococcus | 0.458914 | 0 |
| g__Akkermansia | positive | g__Ruminococcus | 0.363985 | 0 |
| g__Bacteroides | positive | g__Ruminococcus | 0.560511 | 0 |
| g__Bifidobacterium | positive | g__Ruminococcus | 0.412236 | 0 |
| g__Blautia | positive | g__Ruminococcus | 0.339578 | 0.01 |
| g__Clostridium_sensu_stricto_1 | positive | g__Ruminococcus | 0.382021 | 0.02 |
| g__Cutibacterium | positive | g__Ruminococcus | 0.294346 | 0.03 |
| g__Muribaculum | positive | g__Ruminococcus | 0.621633 | 0 |
| g__Parabacteroides | positive | g__Ruminococcus | 0.495353 | 0 |
| g__Romboutsia | positive | g__Ruminococcus | 0.540322 | 0 |
| d__Bacteria | negative | g__Russula | -0.42757 | 0.01 |
| g__Aspergillus | positive | g__Russula | 0.480671 | 0 |
| g__Blautia | negative | g__Russula | -0.33383 | 0.01 |
| g__Clostridium_sensu_stricto_1 | negative | g__Russula | -0.34756 | 0.02 |
| g__Cutibacterium | negative | g__Russula | -0.34606 | 0.02 |
| g__Fusobacterium | negative | g__Russula | -0.36316 | 0.01 |
| g__Inocybe | positive | g__Russula | 0.59269 | 0 |
| g__Lactarius | positive | g__Russula | 0.399644 | 0 |
| g__Rhizopogon | positive | g__Russula | 0.330835 | 0.04 |
| g__Aspergillus | positive | g__Sebacina | 0.323116 | 0.02 |
| g__Bifidobacterium | negative | g__Sebacina | -0.31355 | 0.05 |
| g__Cutibacterium | negative | g__Sebacina | -0.38873 | 0.03 |
| g__Faecalibacterium | negative | g__Sebacina | -0.39328 | 0 |
| g__Fusobacterium | negative | g__Sebacina | -0.42668 | 0 |
| g__Inocybe | positive | g__Sebacina | 0.340835 | 0.04 |
| g__Lactarius | positive | g__Sebacina | 0.471922 | 0 |
| g__Ruhlandiella | positive | g__Sebacina | 0.409312 | 0.01 |
| g__Russula | positive | g__Sebacina | 0.537604 | 0 |
| f__Didymellaceae | positive | g__Tausonia | 0.419899 | 0.01 |
| g__Inocybe | positive | g__Tausonia | 0.389465 | 0.01 |
| g__Rhizopogon | positive | g__Tausonia | 0.365339 | 0.03 |
| f__Didymellaceae | positive | g__unidentified_01 | 0.467244 | 0 |
| f__Lachnospiraceae | negative | g__unidentified_01 | -0.42683 | 0.01 |
| g__Bacteroides | negative | g__unidentified_01 | -0.39529 | 0.02 |
| g__Bifidobacterium | negative | g__unidentified_01 | -0.35885 | 0 |
| g__Blautia | negative | g__unidentified_01 | -0.37906 | 0.01 |
| g__Cladosporium | positive | g__unidentified_01 | 0.560497 | 0 |
| g__Fusicolla | positive | g__unidentified_01 | 0.450722 | 0 |
| g__Inocybe | positive | g__unidentified_01 | 0.74219 | 0 |
| g__Lactarius | positive | g__unidentified_01 | 0.434363 | 0.03 |
| g__Mortierella | negative | g__unidentified_01 | -0.31712 | 0.04 |
| g__Ruhlandiella | positive | g__unidentified_01 | 0.581836 | 0 |
| g__Sebacina | positive | g__unidentified_01 | 0.380659 | 0.02 |
| d__Bacteria | negative | g__unidentified_06 | -0.59867 | 0 |
| g__Blautia | negative | g__unidentified_06 | -0.40506 | 0.02 |
| g__Cutibacterium | negative | g__unidentified_06 | -0.37189 | 0.03 |
| g__Lactarius | positive | g__unidentified_06 | 0.466819 | 0.02 |
| g__Rhizopogon | positive | g__unidentified_06 | 0.298406 | 0.05 |
| g__Ruhlandiella | positive | g__unidentified_06 | 0.455894 | 0 |
| g__Russula | positive | g__unidentified_06 | 0.53808 | 0 |
| g__Sebacina | positive | g__unidentified_06 | 0.311704 | 0.01 |
| g__Blautia | negative | g__Wallemia | -0.40685 | 0.02 |
| g__Cutibacterium | negative | g__Wallemia | -0.36234 | 0.03 |
| g__Prevotella | negative | g__Wallemia | -0.39907 | 0 |
| g__Ruhlandiella | positive | g__Wallemia | 0.611898 | 0 |
| g__unidentified_06 | positive | g__Wallemia | 0.533919 | 0 |
| c__Sordariomycetes | positive | k__Fungi | 0.470634 | 0.02 |
| d__Archaea | positive | k__Fungi | 0.427737 | 0.03 |
| g__Bacteroides | negative | k__Fungi | -0.43026 | 0.02 |
| g__Hygrocybe | positive | k__Fungi | 0.444764 | 0.04 |
| g__Mortierella | positive | k__Fungi | 0.471796 | 0 |
| g__Muribaculum | negative | k__Fungi | -0.5609 | 0.01 |
| g__Parabacteroides | negative | k__Fungi | -0.44276 | 0.03 |
| g__Rothia | positive | k__Fungi | 0.436251 | 0.02 |
| g__Ruminococcus | negative | k__Fungi | -0.35224 | 0.02 |
| f__Didymellaceae | negative | p__Ascomycota | -0.31996 | 0.05 |
| g__Fusicolla | negative | p__Ascomycota | -0.36448 | 0.01 |
| g__Hygrocybe | positive | p__Ascomycota | 0.469816 | 0.01 |
| g__Inocybe | negative | p__Ascomycota | -0.39648 | 0.02 |
| g__Lactarius | negative | p__Ascomycota | -0.40533 | 0.02 |
| g__Mortierella | positive | p__Ascomycota | 0.392643 | 0.02 |
| g__Rothia | positive | p__Ascomycota | 0.381553 | 0.01 |
| g__unidentified_01 | negative | p__Ascomycota | -0.46662 | 0 |
| g__Wallemia | negative | p__Ascomycota | -0.35524 | 0.03 |
| k__Fungi | positive | p__Ascomycota | 0.524256 | 0.02 |
| g__Inocybe | negative | p__Basidiomycota | -0.5171 | 0 |
| g__unidentified_01 | negative | p__Basidiomycota | -0.41033 | 0 |
| p__Ascomycota | positive | p__Basidiomycota | 0.510302 | 0 |
